# Analyzing vegetation health dynamics across seasons and regions through NDVI and climatic variables

**DOI:** 10.1038/s41598-024-62464-7

**Published:** 2024-05-23

**Authors:** Kaleem Mehmood, Shoaib Ahmad Anees, Sultan Muhammad, Khadim Hussain, Fahad Shahzad, Qijing Liu, Mohammad Javed Ansari, Sulaiman Ali Alharbi, Waseem Razzaq Khan

**Affiliations:** 1 College of Forestry, Beijing Forestry University, Beijing, 100083 People’s Republic of China; 2 Key Laboratory for Silviculture and Conservation of Ministry of Education, Beijing Forestry University, Beijing, 100083 People’s Republic of China; 3 Institute of Forest Science, University of Swat, Main Campus Charbagh, Swat, 19120 Pakistan; 4 Department of Forestry, The University of Agriculture, Dera Ismail Khan, 29050 Pakistan; 5 State Forestry and Grassland Administration Key Laboratory of Forest Resources and Environmental Management, Beijing Forestry University, Beijing, 100083 People’s Republic of China; 6 Precision Forestry Key Laboratory of Beijing, Beijing Forestry University, Beijing, 100083 People’s Republic of China; 7 Department of Botany, Hindu College Moradabad (Mahatma Jyotiba Phule Rohilkhand University Bareilly), Moradabad, 244001 India; 8 Department of Botany and Microbiology, College of Science King Saud University, P.O Box 2455, 11451 Riyadh, Saudi Arabia; 9 Department of Forestry Science and Biodiversity, Faculty of Forestry and Environment, Universiti Putra Malaysia, 43400 Serdang, Malaysia; 10 Advanced Master in Sustainable Blue Economy, National Institute of Oceanography and Applied Geophysics - OGS, University of Trieste, 34127 Trieste, Italy; 11 Institut Ekosains Borneo (IEB), Universiti Putra Malaysia Bintulu Campus, 97008 Sarawak, Malaysia

**Keywords:** NDVI, Climatic variability, Vegetation dynamics, Cross wavelet transform, Seasonal variations, Plant sciences, Climate sciences, Environmental sciences

## Abstract

This study assesses the relationships between vegetation dynamics and climatic variations in Pakistan from 2000 to 2023. Employing high-resolution Landsat data for Normalized Difference Vegetation Index (NDVI) assessments, integrated with climate variables from CHIRPS and ERA5 datasets, our approach leverages Google Earth Engine (GEE) for efficient processing. It combines statistical methodologies, including linear regression, Mann–Kendall trend tests, Sen's slope estimator, partial correlation, and cross wavelet transform analyses. The findings highlight significant spatial and temporal variations in NDVI, with an annual increase averaging 0.00197 per year (p < 0.0001). This positive trend is coupled with an increase in precipitation by 0.4801 mm/year (p = 0.0016). In contrast, our analysis recorded a slight decrease in temperature (− 0.01011 °C/year, p < 0.05) and a reduction in solar radiation (− 0.27526 W/m^2^/year, p < 0.05). Notably, cross-wavelet transform analysis underscored significant coherence between NDVI and climatic factors, revealing periods of synchronized fluctuations and distinct lagged relationships. This analysis particularly highlighted precipitation as a primary driver of vegetation growth, illustrating its crucial impact across various Pakistani regions. Moreover, the analysis revealed distinct seasonal patterns, indicating that vegetation health is most responsive during the monsoon season, correlating strongly with peaks in seasonal precipitation. Our investigation has revealed Pakistan's complex association between vegetation health and climatic factors, which varies across different regions. Through cross-wavelet analysis, we have identified distinct coherence and phase relationships that highlight the critical influence of climatic drivers on vegetation patterns. These insights are crucial for developing regional climate adaptation strategies and informing sustainable agricultural and environmental management practices in the face of ongoing climatic changes.

## Introduction

The role of vegetation is irreplaceable in climate regulation, carbon cycle, biodiversity preservation, desertification prevention, and water conservation through photosynthesis and transpiration^1^. Global and regional climate change often affects vegetation growth, which changes its structure and function. The growth status of surface vegetation can also have a positive feedback effect on corresponding climate change^2,3^. As a result, vegetation dynamics and their responses to climate change have become popular topics in global change research^4,5^. Vegetation is vital in preserving climate equilibrium, sustaining the hydrological cycle and carbon equilibrium, and modifying land surface characteristics^6,7^. Terrestrial ecosystems depend significantly on it, and its prevalence is rapidly growing in different regions worldwide, including Pakistan. The intricate and diverse connection between vegetation distribution and factors like climate change, human activities, elevation, soil composition, and other relevant components necessitates in-depth exploration^8^. Researchers have made significant efforts to determine the individual impacts of each factor on vegetation activity at local and global levels^9–12^. The climate plays a central role in regulating the various biological processes of plants. While other non-climatic elements like soil properties, moisture levels, topography, and competition among species, or genetic variability may also impact these cycles^13,14^.

Plant spatial and temporal distribution is influenced by external weather conditions, including precipitation, temperature, and solar radiation^15,16^. For example, temperature changes can affect vegetation processes like photosynthesis and respiration rates. In the Northern Hemisphere, warmer temperatures lead to extended growing seasons and increased plant coverage^17,18^. In contrast, arid regions experience a more significant impact on plant growth due to inadequate precipitation that restricts development. The process of photosynthesis in plants depends on solar radiation as an essential source of heat and has been recognized as a significant factor driving vegetation changes^19^. Moreover, plants impact local or global climate through processes such as regulating surface energy balance, evapotranspiration, and surface water flow^20,21^. Additionally, non-dynamic climate factors such as photoperiod have been found to strongly influence plant phenology across different ecosystem types. Alterations in vegetation dynamics due to abnormal weather conditions can act as a natural experiment when considering potential climate change scenarios^22,23^. Therefore, analyzing spatial and temporal patterns in these instances provides enhanced insight into how Earth's ecosystems adapt to global changes and the potential environmental or economic repercussions. In Pakistan, temperature and precipitation have been identified as crucial influencing factors in various studies on changes in vegetation^24,25^. Mehmood et al.^26^ observed that precipitation and temperature play a role in vegetation's growth and decline. In addition, diminishing surface latent heat flux (downward radiation) has negatively affected vegetation trends in the Khyber Pakhtunkhwa region of Pakistan. Downward radiation serves as the primary energy source for vegetation, while topographic elements also influence soil hydrothermal conditions to some extent. The influences of downward long-wave radiation and topographical factors on changes in vegetation are also significant within mountainous ecosystems.

Studies linking vegetation and climate have limitations. Station-based observations are valuable but provide limited perspectives on broad-scale vegetation shifts^27,28^. The advent of remote sensing technologies has facilitated widespread surveillance to comprehend vegetation dynamics. Satellite data can be used to evaluate vegetation alterations on a large-scale using indices like NDVI and Enhanced Vegetation Index^29,30^. Numerous studies have established a robust association between NDVI and biophysical and biochemical parameters, including leaf area, chlorophyll content, green biomass, and growth conditions. NDVI is the most commonly used vegetation index in about 75% of studies^4^. It represents a normalized contrast between the spectral reflectance in RED and NIR bands, allowing for the analysis of seasonal changes in photosynthetic activity while reducing certain forms of interference (e.g., cloud shadows, topographic effects) often found in individual spectral bands^31–33^.

Cross Wavelet Transform (CWT) analysis is used to study the complex association among different variables in time-series data^34,35^. This analysis enables more precise forecasting and decision-making by detecting data patterns, tendencies, and anomalies^36,37^. Additionally, wavelet analysis can be applied in numerous areas, including finance, environmental, and climate sciences, to gain insight and solve domain-specific challenges^38,39^. Martínez and Gilabert^40^ and Martínez et al.^41^ utilized a wavelet transform-based multi-resolution analysis to examine NDVI time series. The researchers decomposed the series into various temporal scales to capture the vegetation dynamics with intra and inter-annual adjustments. CWT is an effective technique to identify changes associated with vegetation phenology and land-cover changes using precipitation data processed by NDVI images^42^. Similarly, CWT was used to measure the relationship between vegetation indices (NDVI, LAI, VOD) and climatic drivers (precipitation, air temperature, incoming radiation) to explore the different responses of global ecosystems to their climatic change^34,43^.

Situated within the diverse climates of the tropical and subtropical ecological zones, Pakistan has experienced significant climatic impacts on its environmental fabric, particularly in recent years. Despite numerous studies addressing vegetation shifts, a detailed Spatio-temporal examination of vegetation dynamics across Pakistan remains scarce. The country has seen ecological improvements, notably through initiatives like the Billion Tree Tsunami Afforestation Project (BTTAP) (February 9, 2014), significantly benefiting regions like Khyber Pakhtunkhwa, Islamabad, and Punjab. While specific studies have explored the influence of climatic factors on vegetation within restricted areas, employing MODIS-derived vegetation indices, they fall short due to the lack of higher-resolution data from sources like Landsat and Sentinel-based sensors. This gap underscores the need for comprehensive, systematic investigations into the prolonged Spatiotemporal vegetation trends and their climatic interactions across Pakistan across various time frames and spatial dimensions. This study aims to bridge this gap by thoroughly analyzing the shifts in vegetation cover and the climatic drivers behind these changes across different temporal (annual, growing season, seasonal) and spatial (national, zonal, pixel-level) scales. By evaluating the spatial and temporal patterns of vegetation over the past 24 years, quantifying long-term vegetation dynamics, and identifying critical climatic factors such as precipitation, temperature, and solar radiation affecting these patterns, this research endeavors to provide a crucial reference in crafting effective ecological conservation strategies in Pakistan amidst the evolving climate landscape.

## Materials and methods

### Study area

**Figure 1:**
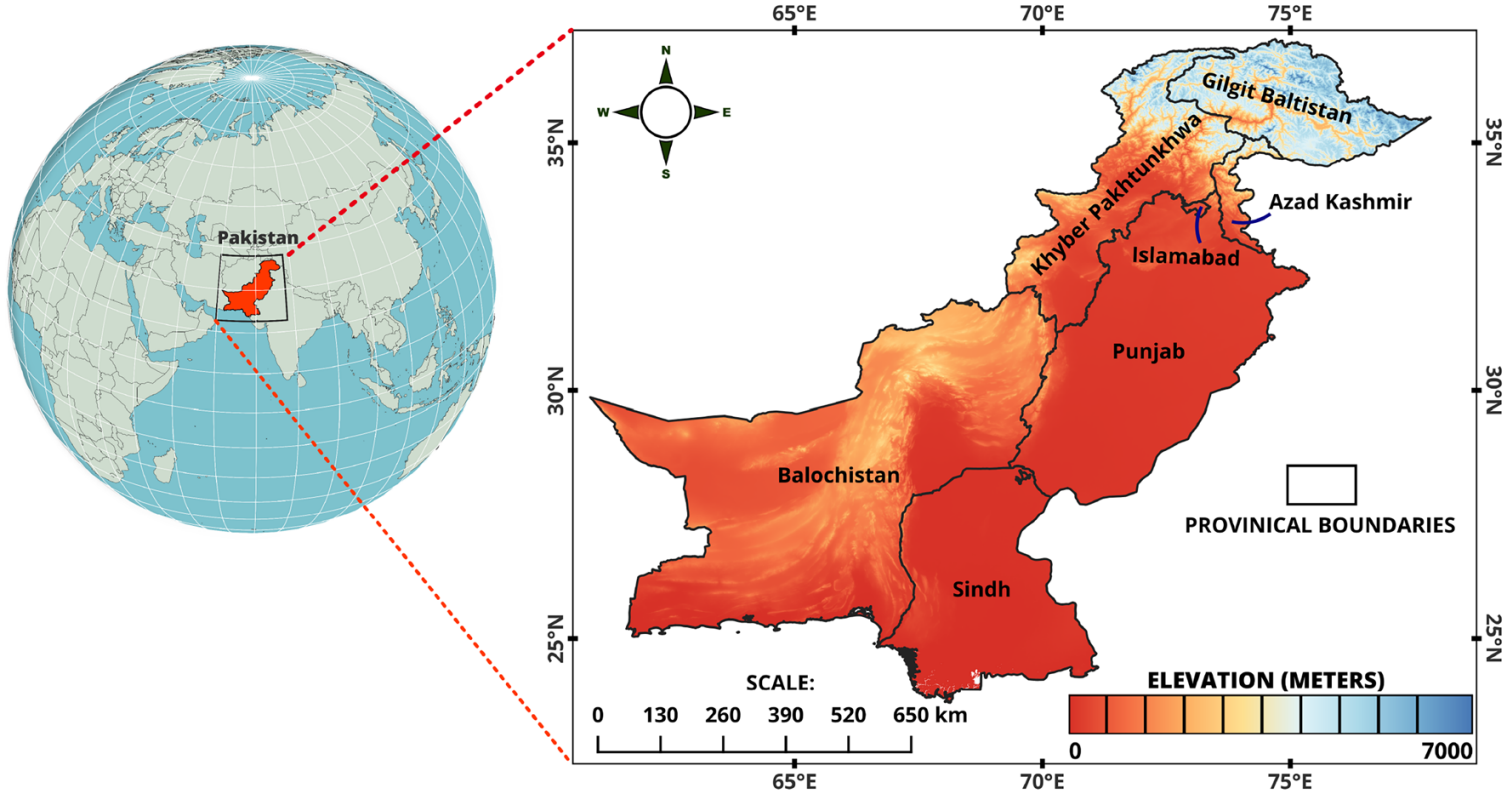
Map of the study area with elevation profile of Pakistan.

The study covered the entirety of Pakistan, a country renowned for its wide-ranging landscapes and climates, leading to a vast array of vegetation types. Pakistan is divided into four provinces—KPK, Punjab (PB), Baluchistan (BL), and Sindh (SD)—as well as unique regions like Azad Jammu and Kashmir (AJK) and Gilgit-Baltistan (GB), each contributing to the country's distinctive environmental traits and ecological diversity. Geographically, Pakistan is bordered by the Arabian Sea to the south, China to the north, India to the east, and Iran and Afghanistan to the west. It also encompasses parts of The Himalayas Mountain range within its boundaries. The country spans from 24° to 37° N latitude and 61° to 77° E longitude, covering an area of approximately 881,913 km^2^ (Fig. 1). Pakistan has diverse forest cover, from mangroves in the south to coniferous forests in the north^44,45^. These forests cover only 5.1% of the land area but are crucial for biodiversity, climate regulation, and livelihoods^46,47^.

The north has moist temperate forests, and the lower zones have tropical deciduous forests^48,49^. The country's soil types vary, reflecting its topography and climate. Alluvial soils are ideal for agriculture, and arid and semi-arid soils dominate the desert regions. Natural resource diversity presents challenges and opportunities for sustainable management and conservation^50,51^. Pakistan's diverse climate significantly impacts its vegetation patterns, with noticeable differences between the north and south. The mountainous northern regions experience chilly winters and pleasant summers, while the southern plains endure hot summers and mild winters. The country has four distinct seasons, including a dry and cool winter from December to February, a hot and dry spring from March to May, a rainy summer season—also known as the southwest monsoon period—from June to September, and a retreating monsoon season in October and November^52–54^. Rainfall varies considerably between the humid coastal areas and the arid conditions in Baluchistan towards the west^55,56^. This study explores the vegetation changes in Pakistan's climates and geographical zones. Pakistan is an ideal research site for vegetation dynamics due to its diverse climate and ecology. We can use analytical techniques like the Mann–Kendall test and cross-wavelet analysis to examine trends and connections between climatic variables and vegetation patterns over time. This provides valuable insights into sustainable management and adaptation strategies for ecological systems and agricultural livelihoods in the face of global climate change.

### Data source

#### NDVI analysis: leveraging high-resolution landsat data and cloud computing

Our study adopts the high-resolution Landsat series for NDVI derivation, deviating from traditional coarse-resolution methods. This approach enhances spatial and temporal analysis depth despite higher computational demands. We addressed computational challenges by segmenting the study area into province-based units and utilizing Google Earth Engine (GEE) for efficient data processing. The final analysis was conducted in R, ensuring a detailed and comprehensive vegetation dynamics study. We used the maximum value composite technique to deal with interferences from clouds, atmosphere, and solar zenith angle in our Landsat-derived NDVI dataset. This method, as described by^57,58^ involves selecting the highest monthly NDVI value for each pixel to minimize environmental noise. Subsequently, we calculated average monthly NDVI values from 2000 to 2023: yearly, growing season, and standard meteorological seasons to accurately analyze vegetation dynamics.

#### Climate data

**Figure 2:**
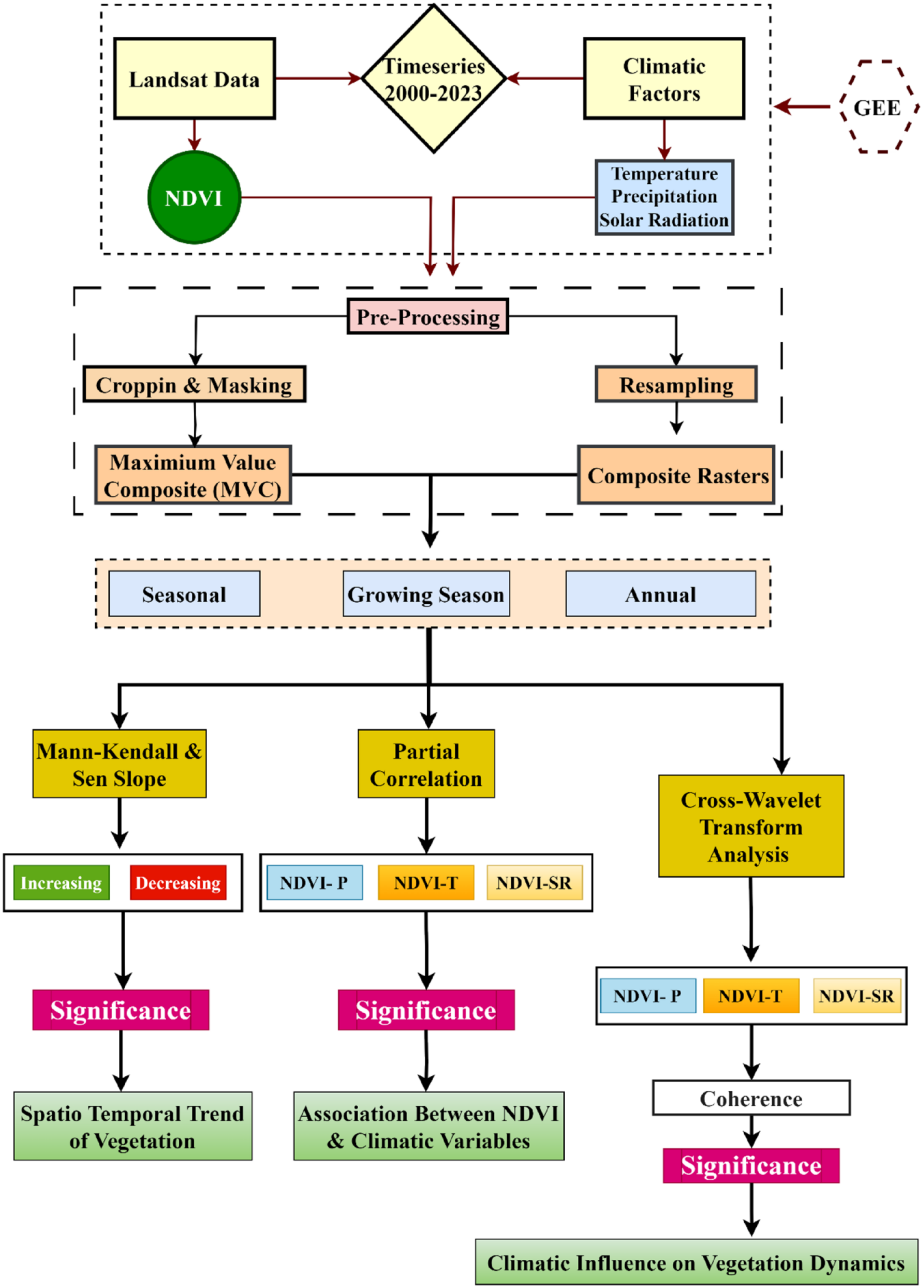
Methodological flowchart for analyzing vegetation dynamics and climatic influences using remote sensing data (2000–2023).

The study utilized average monthly continuous data spanning 24 years (2000–2023). We collected monthly precipitation (*mm*), from the CHIRPS Dataset. The CHIRPS data product is a cutting-edge tool developed by the US Geological Survey Earth Resources Observation and Science Center in partnership with the Santa Barbara Climate Risk Group at the University of California^59,60^ . It provides up-to-date information on precipitation spanning from 1981 to the present day, covering an area from 50° S to 50° N and from 180° E to 180° W. With a spatial resolution of 0.05° (5 km) and daily, pentad, and monthly temporal resolution, CHIRPS is specifically designed to monitor drought conditions in areas with complex topography and deep precipitation systems^59,61^. For this study, we used the GEE platform to download the CHIRPS data product (Precipitation, mm) for Pakistan from 2000 to 2023. In addition, we extracted solar radiation (*SR*, W/m^2^), and average mean temperature (°C) data at a height of 2 m from the ERA5 Reanalysis datasets^62,63^. The ERA5 datasets offer a comprehensive historical archive from 1958 to the present and are characterized by their acceptable spatial resolution of 9 km^64,65^. To ensure a consistent and accurate comparison and analysis across different data types, we aligned all gathered climate data to match the spatiotemporal resolution of the NDVI data through a resampling process^66,67^. The overall schematic methodology of the research is represented in Fig. 2.

#### Vegetation types

**Figure 3:**
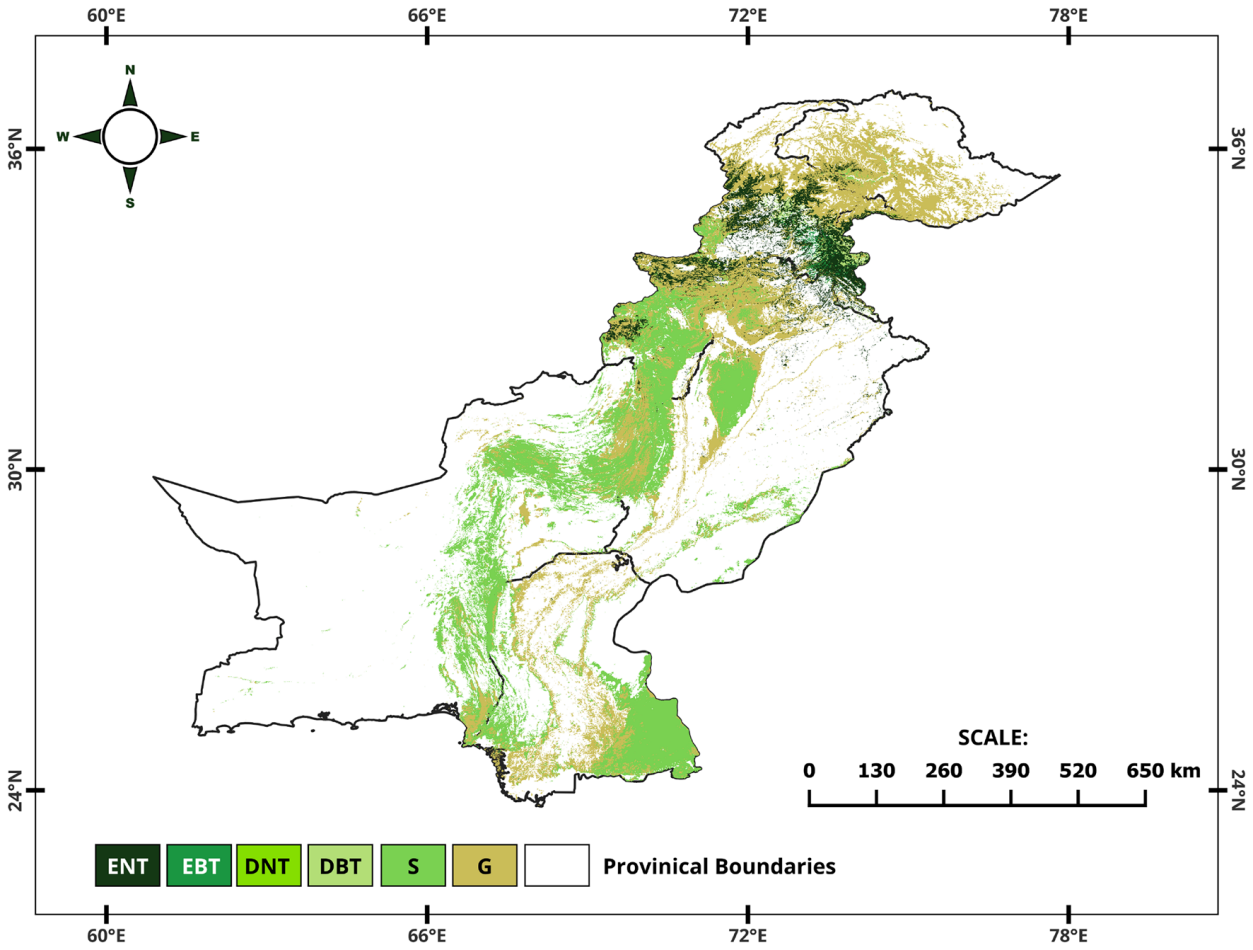
Distribution of vegetation types in Pakistan.

For this research, we utilized the MODIS Land Cover Type 5 dataset, classifying global vegetation into 11 categories based on plant functional types^68–70^. Our focus was on analyzing the dynamics of vegetation in Pakistan, so we chose eight classes that represent pure vegetation types, including Evergreen Needleleaf Trees (ENT), Evergreen Broadleaf Trees (EBT), Deciduous Needleleaf Trees (DNT), Deciduous Broadleaf Trees (DBT), Shrub (S), and Grass (G) (Fig. 3). By analyzing the coverage area and changes in these classes over time, we could reasonably understand and compare the distribution and temporal changes of vegetation types in Pakistan. With the dataset's high spatial resolution and annual update frequency, we could conduct a detailed and dynamic vegetation analysis, contributing to our knowledge of the country's environmental changes and vegetation trends. We excluded non-vegetative and mixed classes such as urban areas, water bodies, and non-vegetated lands to ensure a focused study on natural vegetation dynamics.

### Methodology

#### Interannual vegetation dynamics analysis in Pakistan: a linear regression approach

To evaluate changes in vegetation and climate patterns in Pakistan annually, we created a linear regression model that links the *NDVI*_*i*_ with changes over time. The model’s parameters were determined using the least squares approach^71,72^, utilizing the equations that follow:

1 $$slope= \frac{n \times \sum_{i=1}^{n}\left(i \times {NDVI}_{i}\right)- \sum_{i=1}^{n}i\sum_{i=1}^{n}{NDVI}_{i}}{n \times \sum_{i=1}^{n}{i}^{2}- {\left(\sum_{i=1}^{n}i\right)}^{2}}$$

Equation (1) is used, where slope denotes the rate of change, and *NDVI*_*i*_ represents the variable connected with the *i*th observation. A positive slope *(slope* > *0)* indicates an increase in vegetation dynamics, while a negative slope *(slope* < *0)* implies a decline. The statistical significance of the model was evaluated using an *F*-test, detailed as Eq. (2):

2 $$F= \frac{SSR}{SSE/\left(n-2\right)}$$

where *SSR* is the sum of squares due to regression. *SSE* is the sum of squares. The degrees of freedom for the residuals are 2n-2, where n is the number of observations. The estimated NDVI values derived from the linear regression model are represented by *NDVI*_*i*_. The F-test determines the statistical significance of vegetation trends over time, with *p-values* less than 0.05, 0.01, and 0.001 indicating significant, more significant, and highly significant levels, respectively.

#### Trend analysis

##### Mann–Kendall trend analysis

The Mann–Kendall (MK) test is a highly reliable non-parametric approach to trend analysis^73,74^. Its ability to operate independently of specific data distributions and tolerance for outliers stands out. Utilizing a standardized Z test statistic can effectively evaluate trends without making any assumptions about the underlying data distribution—the S statistic and its variance serve as the essential formula for trend detection and assessment^75,76^. The S statistic is calculated by combining the results of the signum function for all data pairs *(x*_*j*_* − x*_*i*_*),* where xj​ and xi​ are data values at different points in time and n is the total number of data points (Eq. 3). The signum function, *sgn(x*_*j*_* − x*_*k*_*),* assigns a value of + 1, 0, or − 1 depending on the comparison result between data points xj​ and xk​ (Eq. 4).

To effectively analyze trends in environmental data, like NDVI measurements, we use a methodology that quantifies trend direction and magnitude through the Mann–Kendall test and Sen's slope estimator. This process considers ties within the dataset to calculate the variance of S, Var(S), which is used to compute the standardized test statistic Z^77^ (Eq. 5). A negative Z indicates a downward trend. In contrast, a positive Z indicates an upward trend. A statistically significant 5% confidence level trend is signal led by a Z value beyond ± 1.96, as shown in (Eq. 6).

3 $$S=\sum_{k=1}^{n-1}\sum_{j=k+1}^{n}sgn\left({x}_{i}-{x}_{j}\right)$$

4 $$sgn\left({x}_{j}-{x}_{k}\right)= \left\{\begin{array}{ll}+1 &\quad when \left({x}_{j}-{x}_{k}\right)>0\\ 0 &\quad when \left({x}_{j}-{x}_{k}\right) =0\\ -1 &\quad when \left({x}_{j}-{x}_{k}\right)<0\end{array}\right.$$

5 $$Var\left(S\right)= \frac{n\left(n-1\right)\left(2n+5\right)- \sum_{p=1}^{q}tp \left(tp-1\right)(2tp+5)}{18}$$

6 $$Z=\left\{\begin{array}{ll} \frac{s-1}{\sqrt{var (s)}} &\quad when S>0\\ 0 &\quad when S=0\\ \frac{s+1}{\sqrt{var (s)}} &\quad when S<0\end{array}\right.$$

##### Sen slope estimator

The Sen's Slope Estimator is a non-parametric approach for calculating the slope of a trend^78–80^, which reveals the yearly shift and is ideal for linear trends that don't depend on any assumptions about data distribution^81^. *Qi* is obtained by taking the median of the discrepancies between xj and xk, as well as data values at times j and k (Eq. 7). Sen's slope *(Q*_*med*_) is the median value for an odd number of data points *N* (Eq. 8), whereas, for an even N, it's the average of the middle two values (Eq. 9).

7 $${Q}_{i}= \frac{{x}_{j}-{x}_{k}}{j-k} for i=1,\dots , N$$

8 $${Q}_{med}={Q}_{\left[\left(n+1\right)/2\right]}$$

9 $${Q}_{med}=\frac{1}{2}\left({Q}_{\left[N/2\right]}+ {Q}_{\left[\left(N+2\right)/2\right]}\right)$$

10 $${C}_{{\alpha }}={Z}_{1-\frac{\alpha }{2}} \sqrt{Var (S)}$$

To determine the confidence interval for the true slope at a significance level, we use standard normal distribution values denoted as Z(1-α/2) and the variance of the slope (Eq. 10). The ordered slope estimates, Q_i_, are used to calculate the lower and upper confidence limits, Q_min_ and Q_max_, while considering M1 = (N-Cα​)/2 and M2 = (N + Cα)/2. Interpolation is applied if necessary for non-integer M1 and M2^82–84^.

#### Partial correlation analysis

The correlation between vegetation dynamics and individual climatic factors can be challenging to measure accurately due to irrelevant variables^85^. As a result, correlation coefficients may not fully reflect the true extent of the correlation^86,87^. To address this issue, we employed second-order Pearson partial correlation analysis, which allows us to isolate the influence of two additional variables. This method provides a more refined examination of the correlation between NDVI and specific climatic factors. The computational methodologies for determining the partial correlation coefficient can be outlined as follows:

11 $${P}_{mn}= \frac{\sum_{i=1}^{n}\left[\left({m}_{i}-\overline{m }\right)\left({n}_{i}-\overline{n }\right)\right]}{\sqrt{\sum_{i=1}^{n}{\left({m}_{i}-\overline{m }\right)}^{2}} \sqrt{\sum_{i=1}^{n}{\left({n}_{i}- \overline{n }\right)}^{2}}}$$

where *n* is the number of observations, *mi*​ and *ni*​ are the individual samples of variables m and n. m and n​ are the mean values of variables m and n, respectively. The Partial Correlation Coefficient, *P*_*m n, o*_, controlling for the effect of variable *o* is defined as:

12 $${P}_{mn,o}= \frac{{P}_{mn}-{P}_{mo}\times {P}_{no}}{\sqrt{\left(1-{P}_{mo}^{2}\right)\left({1-P}_{no}^{2}\right)}}$$

Here, *P*_*mn*_​, *P*_*mo*_​, and *P*_*no,*_​ represent the Pearson correlation coefficients between the respective variable pairs. The Extended Partial Correlation Coefficient, *P*_*mn,op​,*_ controlling for the effects of two variables, *o* and *p*, is calculated as:

13 $${P}_{mn,op}= \frac{{P}_{mn,o}- {P}_{mp,o}{P}_{np,o}}{\sqrt{\left({P}_{mp,o}^{2}\right)\left({P}_{np,o}^{2}\right)}}$$

In this formula, *P*_*mn,o​,*_* P*_*mp,o*_ and *P*_*np,o,*_​ denote the partial correlation coefficients between the respective variable pairs, with the effect of variable *o* accounted for. The significance of the partial correlation coefficient can be tested using a t-test, which is formulated as follows:

14 $$\text{t}=\sqrt[P]{\frac{n-q-2}{{1-P}^{2}}}$$

where *P* represents the partial correlation coefficient being tested (*P*_*mn,o*_​ or *P*_*mn,op*_​). *n* is the total number of observations. *q* is the number of controlled variables (*q* = 1 for *P*_*mn,o*_​ and *q* = 2 for *P*_*mn,op*_​). *t* is the test statistic used to determine the significance of the correlation based on degrees of freedom (*n* − *q* − 2). The t-value can be compared to critical values from the t-distribution to evaluate the statistical significance of the partial correlation with a predetermined alpha level (0.05 or 0.01).

#### Cross-wavelet transform analysis of NDVI and climate interactions

Continuous wavelet transform (CWT) involves converting a time-series signal, denoted as *X*_*t*_, into the time–frequency domain, allowing for practical signal magnitude and periodicity analysis^88–90^. To achieve this, the Morlet wavelet is utilized by convolving *X*_*t*_ with "wavelet daughters," scaled and translated versions^88,91^. This transformation is mathematically defined as follows (Eq. 15):

15 $$Wave \left(\tau ,s\right)= {\sum }_{t}{x}_{t} \frac{1}{\sqrt{s}}\Psi \star \left(\frac{t-\tau }{s}\right)$$

where *s* is the scale of the wavelet, *τ* is the wavelet's translation in time, Ψ is the mother Morlet function and ∗ indicates the complex conjugate. This analysis utilizes the Morlet wavelet, denoted as $\Psi$ (t), as the foundational wavelet function^92,93^. Its formula is as follows (Eq. 16):

16 $$\Psi \left(t\right)= {\pi }^{-1/4}{e}^{-i\omega t}{e}^{{-t}^{2}/2}$$

Here, ω denotes the angular frequency in radians per unit time, and t represents the time step. A complete cycle's period (inverse frequency) is equivalent to 2π radians, measured as 2π/ω ​. This scenario implicitly defines the time-scale period with ω set to 6. The wavelet power formula can determine the energy present at each scale and translation in the wavelet transform (Eq. 17).

17 $$Power\left(\tau ,s\right)=\frac{1}{s} {\left|Wave\left(\tau ,s\right)\right|}^{2}$$

This approach enables us to gain insight into the relationship between NDVI and climatic variables over comparable time intervals by conducting a CWT analysis. Such analysis provides a holistic perspective of their connections. The cross-wavelet analysis combines two-time series, wx(τ,s) and wy(τ,s) (where the asterisk denotes the complex conjugate), to produce the cross-wavelet coefficient^94^ (Eq. 18).

18 $${W}_{xy}\left(\tau ,s\right)={\omega }_{x}\left(\tau ,s\right).{\omega }_{y}\star \left(\tau ,s\right)$$

This coefficient calculates the coherence, a measure of the correlation between the two-time series (Eq. 19 and Eq. 20).

19 $$Coherence= \frac{{\left|s.Wave.xy\right|}^{2}}{sPower.x . sPower.y}$$

20 $$Power.x= \frac{1}{s}{\left|Wave\left(\tau ,s\right)\right|}^{2};Power.y= \frac{1}{s}{\left|Wave \left(\tau ,s\right)\right|}^{2}.$$

Coherence is determined by taking the squared magnitude of the cross-wavelet coefficient and dividing it by the product of the power spectra of the individual time series. The arrows in the resulting plots can infer phase differences between the time series representing the leading or lagging relationships between the variables^95,96^. Coherence was assessed at a significance level of 0.05 within the cone of influence using the *WaveletComp* package in *R*^97,98^.

## Results

### Climate and vegetation dynamics across Pakistan

Regional environmental parameters across various regions of Pakistan were summarized in this analysis, with particular emphasis on NDVI, precipitation, temperature, and SR, which are essential in understanding climate impacts and forest dynamics. The average NDVI for Pakistan at the national level is 0.2345 ± 0.139, and the mean precipitation and temperature are 477.64 ± 357.38 mm and 14.8155 ± 13.18 °C, respectively (Table 1). Pakistan's average NDVI suggests moderate vegetation health across the country, influenced by various regional climatic conditions. The significant standard deviations in precipitation and temperature at the national level indicate a wide variability across the regions, underlining the diverse climatic zones within Pakistan. Solar radiation averages are moderately consistent across areas and do not show drastic differences, suggesting that sunlight distribution is not a major distinguishing factor among these areas.

**Table 1:** Statistics of annual NDVI and important climate variables.

Research areas	NDVI	P (mm)	T (°C)	SR (W/m^2^)
KPK	0.267 ± 0.02	641.38 ± 88.15	11.918 ± 0.538	220.403 ± 3.497
AJK	0.433 ± 0.023	1119.07 ± 157.45	11.181 ± 0.498	210.948 ± 3.696
GB	0.061 ± 0.007	341.7 ± 47.46	-8.421 ± 0.556	241.306 ± 2.591
Baloch	0.102 ± 0.01	163.75 ± 46.86	22.24 ± 0.481	249.893 ± 4.021
Punjab	0.326 ± 0.027	400.61 ± 71.36	24.842 ± 0.489	224.249 ± 4.012
Sindh	0.218 ± 0.018	199.38 ± 98.68	27.133 ± 0.322	239.966 ± 4.116
Pakistan	0.2345 ± 0.139	477.64 ± 357.38	14.8155 ± 13.18	231.1275 ± 13.18

The AJK region has the highest NDVI value of 0.433 ± 0.023, indicating robust vegetation, likely due to substantial precipitation recorded at 1119.07 ± 157.45 mm. This high level of precipitation, although beneficial for vegetation, suggests a significantly wetter climate compared to other areas. Despite this, the temperature in AJK maintains a mean similar to KPK, at approximately 11 °C, indicating a mild climate conducive to vegetation growth but not excessively warm. BL defects different scenarios as it has a relatively low NDVI (0.102 ± 0.01) and the highest average temperature (22.24 ± 0.481 °C) among the surveyed regions. The temperature seems crucial in delaying vegetation growth, potentially due to excessive heat and inadequate water supply, despite its lower precipitation (163.75 ± 46.86 mm). PB, on the other hand, displays a more favorable balance between temperature (24.842 ± 0.489 °C) and precipitation (400.61 ± 71.36 mm), which is reflected in a higher NDVI (0.326 ± 0.027). This indicates that despite higher temperatures compared to other regions, sufficient precipitation supports better vegetation conditions.

SD shows moderate vegetation health, with an NDVI of 0.218 ± 0.018, which may be attributed to its intermediate precipitation level (199.38 ± 98.68 mm) and the highest average temperature (27.133 ± 0.322 °C). The relatively higher temperature could mitigate precipitation's positive impact on vegetation. The analysis of monthly and seasonal trends in KPK and AJK shows that the NDVI attains its highest point in July and August, indicating significant vegetation growth initiated by the monsoon rains. On the other hand, in GB, the vegetation experiences its peak in July, owing to the short summer period. The arid climate of BL results in a moderate increase in NDVI during the same monsoon months, which highlights the relative impact of the season even in less humid areas. Conversely, PB and SD witnessed a decline in NDVI during April, May, and June, probably due to the harvesting season and the effect of agricultural activities (Figure S1). These observations highlight the complex association between weather patterns, climate, and human practices, with specific months like July and August being significant for the growth of natural and cultivated vegetation in various regions.

### Interannual and seasonal trends of NDVI and climate predictors

**Figure 4:**
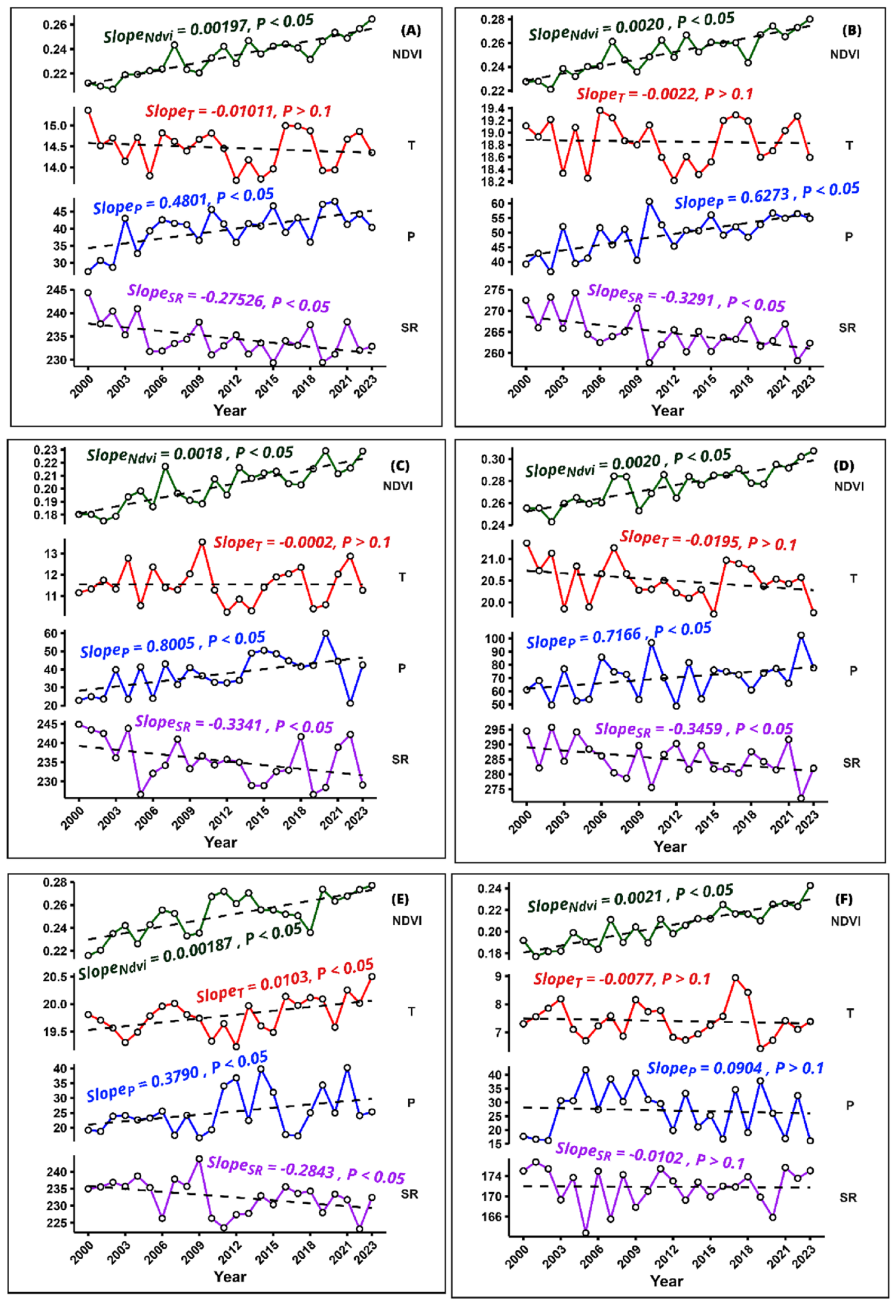
Temporal dynamics of NDVI and principal climatic predictors across various temporal scales within Pakistan (2000–2023): (**A**) annual overview; (**B**) growing season; (**C**) Spring; (**D**) summer; (**E**) autumn; (**F**) winter.

In Pakistan, the NDVI and precipitation exhibit significant annual increases, with slopes of 0.00197 and 0.4801 mm yr^−1^, respectively (NDVI p < 0.0001, precipitation p = 0.0016). However, the yearly temperature trend displays a minor decline with a slope of − 0.01011 °C yr^−1^, which is not statistically significant (p = 0.465). Similarly, the annual solar radiation undergoes a significant decrease, with a slope of − 0.27526 W m^−2^ yr^−1^ (p = 0.011). These trends imply a significant transformation in environmental parameters, which could have important implications for the local ecosystem and climate dynamics (Fig. 4A). Environmental changes were also observed during the autumn season (Fig. 4E). The NDVI showed a substantial increase with a slope of 0.0018792 years^−1^, marking a highly significant upward trend (p < 0.0001). Temperature also displayed a notable rise, with a slope of 0.023194 °C yr^−1^, indicating a statistically significant warming trend (p = 0.0103). Precipitation trends were positive, although slightly significant, with a slope of 0.3790 mm yr^−1^ (p = 0.0706), while solar radiation displayed a marginally significant decreasing trend with a slope of − 0.2843 W m^−2^ yr^−1^ (p = 0.0559). Considerable changes were observed during the growing season (GS) (Fig. 4B).

The NDVI showed a significant upward trend with a slope of 0.0020 years − 1 (p < 0.0001). However, the temperature experienced a slight decline at a rate of -0.0022 °C yr^−1^, but this change was not statistically significant (p = 0.842). Precipitation significantly increased, with a slope of 0.6273 mm yr^−1^ (p = 0.00022), while solar radiation decreased significantly, with a slope of − 0.3291 W m^−2^ yr^−1^ (p = 0.00784). In the spring, there was a highly significant increase in NDVI, with a slope of 0.0018 years^−1^ (p < 0.0001) (Fig. 4C). Temperature trends were stable, showing an insignificant decrease of − 0.0002 °C yr^−1^ (p = 0.995). Precipitation significantly ascended, with a slope of 0.8005 mm yr^−1^ (p = 0.0057), and solar radiation displayed a significant decline, with a slope of − 0.3341 MJ m^2^ yr^−1^ (p = 0.0478). Over the summer, a substantial rise in NDVI was observed with a slope of 0.0020 years^−1^ (p < 0.0001) (Fig. 4D). Temperature slightly decreased by − 0.0195 °C yr^−1^, which was insignificant (p = 0.149). Precipitation exhibited a positive but marginally significant trend with a slope of 0.7166 mm yr^−1^ (p = 0.0852), and solar radiation decreased significantly, with a slope of − 0.3459 W m^−2^ yr^−1^ (p = 0.0487). In winter, the NDVI continued to increase substantially with a slope of 0.0021 years^−1^ (p < 0.0001) (Fig. 4F). Temperature and precipitation changes were minor and not statistically significant, with slopes of − 0.0077 °C yr^−1^ (p = 0.681) and − 0.0904 mm yr^−1^ (p = 0.725), respectively. Solar radiation exhibited a negligible decrease with a slope of − 0.0102 W m^−2^ yr^−1^ (p > 0.1).

### Regional variations in NDVI patterns: insights from Mann–Kendall trend

**Figure 5:**
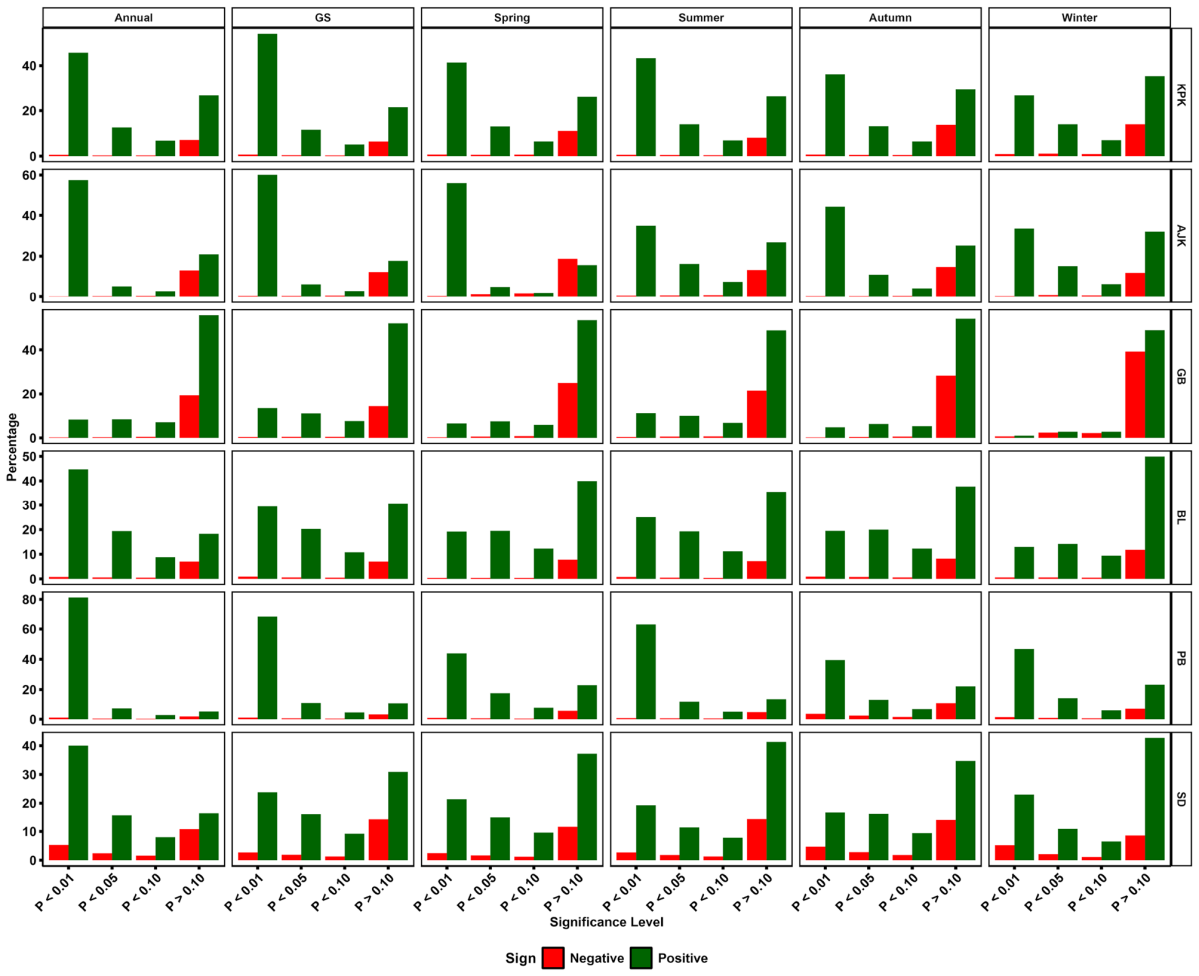
Percentage analysis of NDVI Mann–Kendall trend across various regions (KPK, AJK, GB, BL, PB, SD) and seasons (annual, GS, Spring, summer, autumn, winter). This plot shows the Mann–Kendall trend results, indicating changes in vegetation index at statistical significance levels (P < 0.01, P < 0.05, P < 0.10, and P > 0.10).

**Figure 6:**
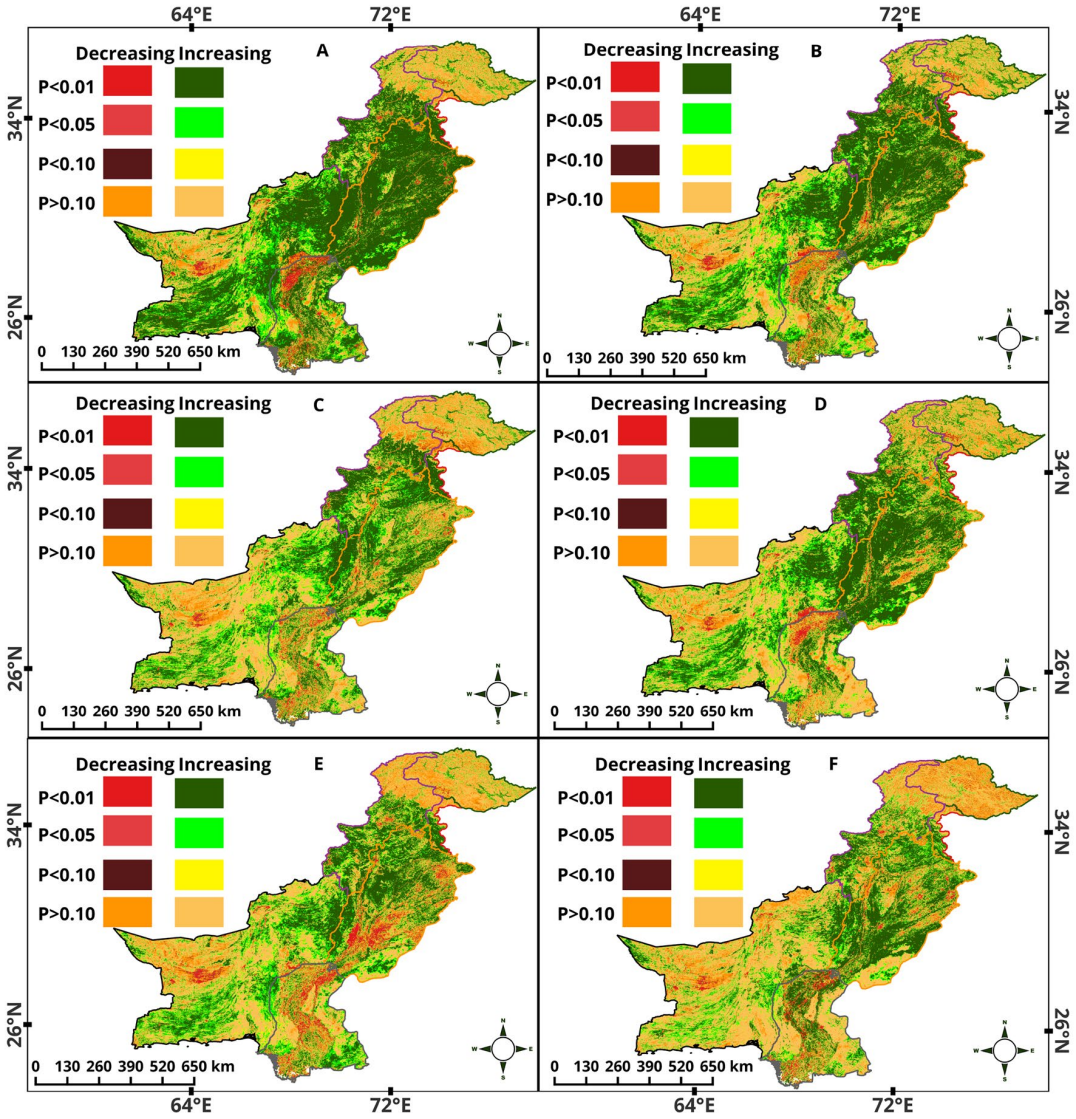
Spatial distribution of NDVI trends in Pakistan in (**A**) annual, (**B**) growing season, (**C**) spring, (**D**) summer, (**E**) autumn, and (**F**) winter. Each color represents a different threshold of significance level (%).

Distinct variations in environmental conditions were observed across Pakistan's provinces annually, as evidenced by the results of the Mann–Kendall test. KPK showed a remarkable greening trend, with 45.7% of its area displaying a significant increase in NDVI values at p < 0.01. This increase indicated a substantial improvement in vegetation health on an annual basis (Fig. 6A). Only 0.6% of the area showed a significantly decreased NDVI, pointing to overall positive ecological growth across the province and contrasting with the greening trend Fig. 6B). In AJK, the annual increase was even more noticeable, with 57.5% of the region showing significant positive changes (Table S4). This finding suggested improvements in vegetation conditions. GB and BL showed more modest positive changes annually at significant levels (p < 0.01) of 8.3% and 44.7%, respectively, indicating regional disparities in environmental trends. PB and SD emerged as areas of significant ecological interest, with 81.2% and 40.0% of their territories exhibiting significant annual vegetation increases. This finding reflected substantial greenery and environmental enhancement, particularly in Punjab. During the GS, KPK and AJK continued leading in significant vegetation increases, observed in 54.0% and 60.1% of their lands, respectively. At the same time, GB and BL showed less pronounced trends than the annual data, emphasizing the seasonal variances in ecological health (Fig. 5).

Punjab maintained its ecological robustness, with 68.2% of its area displaying significant positive changes, while Sindh showed a greening trend in 23.8% of its territory during the GS. The data provide a detailed picture of seasonal ecological dynamics. Spring resulted in significant positive changes across all provinces, with the highest in AJK (56.04%) and the lowest yet considerable in GB (6.55%), highlighting the season's critical role in vegetation recovery and growth (Fig. 6C). The summer trends were particularly noteworthy in Punjab, where 63.0% of the area experienced significant vegetation increases, underscoring the summer's crucial impact on agricultural and natural ecosystems (Fig. 6D). During the winter and autumn season, they showcased a more balanced increase across regions. KPK, AJK, and PB showed notable increases in NDVI values, indicative of winter's varying influence on vegetation health across different climatic zones (Fig. 6F & 6E). The ecological landscape in Pakistan is generally positive but complex, with seasonal fluctuations and regional variations. Customized environmental strategies are needed to maintain and improve the ecological well-being of the diverse landscapes.

### Regional and seasonal NDVI responses to climatic variables in Pakistan

**Figure 7:**
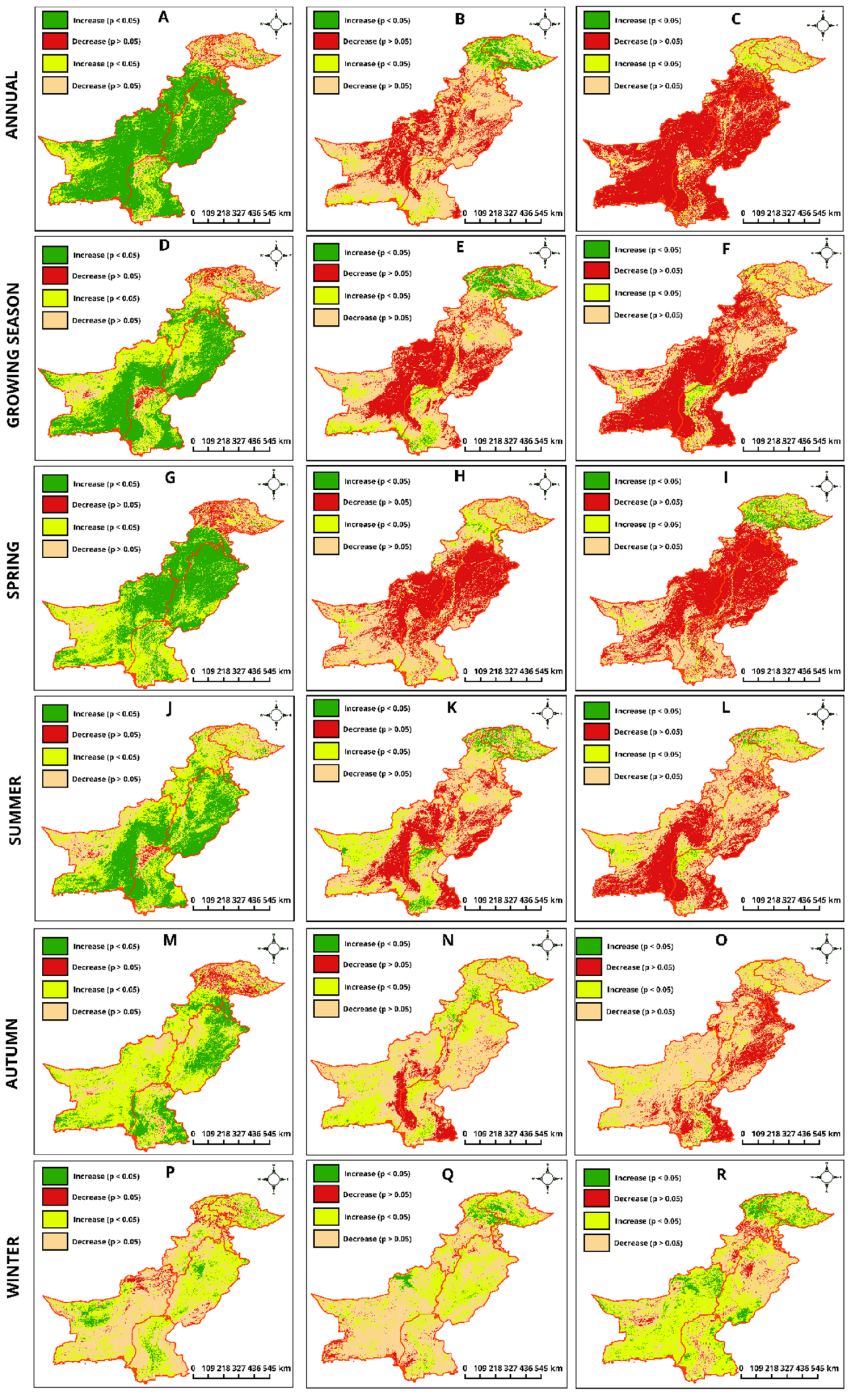
Spatial distribution of partial correlation coefficients between NDVI and climatic variables in Pakistan. The correlation between NDVI and precipitation, temperature, and solar radiation (SR) is represented across different temporal scales: annual (**A**–**C**), growing season (**D**–**F**), Spring (**G**–**I**), Summer (**J**–**L**), Autumn (**M**–**O**), and Winter (**P**–**R**). Each color represents different threshold of significance level (%).

In a comprehensive analysis of NDVI responses to climatic variables across Pakistan, distinct regional and seasonal patterns emerge. In KPK, there is a nuanced annual interaction between NDVI and precipitation (50.2% showing significant change), contrasting with its reaction to temperature, where a balanced significant response is less pronounced (15.07% positive versus 20.4% negative). This suggests a complex hydro-thermal dynamic affecting vegetation. However, non-significant trends cover 18.7% of the area, indicating areas where precipitation less directly influences NDVI (Table 2). Contrastingly, AJK exhibits a more even distribution between significant positive and negative responses to precipitation annually, with a notable shift towards non-significant trends in warmer months, hinting at varied vegetation sensitivity across seasons (Fig. 7D–F). GB stands out with minimal significant responses to precipitation but shows a considerable reaction to temperature changes (39.1% positive), indicating temperature might be a more dominant factor influencing vegetation in this high-altitude region (Fig. 7P–R). In BL, a substantial portion of the area demonstrates significant annual responses to precipitation (76.1%), aligning with the arid climate's expected dependency on water availability for vegetation growth (Fig. 7M–O). However, this region, alongside PB, reveals minimal significant positive responses to temperature changes annually, underlining potential adaptive mechanisms or other prevailing environmental factors diluting temperature's direct impact on NDVI. Sindh showcases a unique scenario with a moderately significant positive response to precipitation annually (55.1%). Still, it exhibits less sensitivity to temperature variations, reflected in the minimal significant positive change (0.8%) (Fig. 7A–C). This could point towards water as a limiting factor rather than thermal conditions for SD 's vegetation health. Furthermore, SR's impact presents a diverse picture; regions like KPK and AJK show negligible significant positive responses annually, suggesting potential limitations due to water stress or cloud cover, particularly during peak sun exposure periods. Conversely, BL overwhelming non-significant negative response (85.4%) to SR annually indicates a potential for overexposure leading to vegetation stress, a situation mirrored albeit to a lesser extent in PB and SD.

**Table 2:** Percentage of area exhibiting NDVI responses to climatic variables across various timescales in Pakistan.

Parameters	Research zone		Annual	GS		Spring	Summer	Autumn	Winter	
NDVI & P (mm)	KPK	(↑↓)	(50.2, 8.35)	(22.7, 5.97)		(58.0, 14.4)	(15.6, 1.18)	(16.1, 13.1)	(2.58, 10.7)	
		(↗↘)	(18.7, 22.6)	(53.7, 17.5)		(11.5, 16.0)	(64.6, 18.5)	(47.7, 22.8)	(35.6, 51.1)	
	AJK	(↑↓)	(47.1, 10.1)	(45.9, 2.68)		(52.4, 17.7)	(25.8, 1.49)	(60.9, 9.63)	(1.95, 14.8)	
		(↗↘)	(12.3, 30.3)	(26.2, 25.1)		(10.0, 19.6)	(49.5, 23.1)	(18.5, 10.8)	(49.5, 33.6)	
	GB	(↑↓)	(4.70, 16.5)	(7.19, 21.4)		(1.51, 27.8)	(4.77, 3.55)	(2.02, 28.7)	(9.55, 7.84)	
		(↗↘)	(20.7, 58.4)	(20.6, 50.7)		(17.8, 52.7)	(41.7, 49.9)	(22.4, 46.8)	(53.9, 28.6)	
	BL	(↑↓)	(76.1, 0.22)	(47.5, 1.08)		(38.6, 0.26)	(39.3, 2.17)	(8.62, 0.4)	(4.61, 5.43)	
		(↗↘)	(21.6, 2.05)	(41.2, 10.1)		(53.8, 7.19)	(36.1, 22.3)	(71.9, 19.1)	(30.63, 59.33)	
	PB	(↑↓)	(87.1, 0.59)	(64, 0.41)		(77.8, 0.26)	(59.1, 0.16)	(35.7, 0.10)	(5.57, 1.52)	
		(↗↘)	(10.3, 2)	(31.3, 4.30)		(19.8, 2.04)	(36.7, 3.87)	(53.1, 11.1)	(57.1, 35.8)	
	SD	(↑↓)	(55.1, 1.69)	(47.1, 6.16)		(25.9, 0.58)	(43.5, 4.81)	(38.5, 2.07)	(6.39, 0.57)	
		(↗↘)	(34, 9.18)	(33.8, 12.7)		(62.7, 10.8)	(40.1, 11.5)	(42.6, 16.7)	(41.7, 51.3)	
NDVI & T (°C)	KPK	(↑↓)	(15.0, 20.4)	(15.8, 20)		(3.86, 15.9)	(8.72, 15.1)	(6.75, 2.26)	(9.21, 4.69)	
		(↗↘)	(18.8, 45.6)	(13.8, 50.3)		(28, 52.1)	(17.3, 58.8)	(53.8, 37.1)	(33.4, 52.6)	
	AJK	(↑↓)	(19.7, 17.7)	(18.3, 13, 3)		(8.32, 3.98)	(9.50, 4.22)	(1.43, 1.61)	(14.5, 16.2)	
		(↗↘)	(22.7.39.8)	(15.9, 52.3)		(37.4, 50.2)	(19.9, 66.3)	(55.8, 41.7)	(24.2, 44.9)	
	GB	(↑↓)	(39.1, 0.7)	(40.7, 2.2)		(1.78, 9.76)	(26.6, 5.08)	(5.12, 1.80)	(16.8, 2.04)	
		(↗↘)	(38.3, 21.7)	(38.2, 18.7)		(29.4, 59.1)	(39.5, 28.71)	(52.4, 40.6)	(54.3, 26.7)	
	BL	(↑↓)	(0.2, 35.8)	(1.1, 45.8)		(0.32, 41.8)	(1.58, 31.6)	(0.72, 9.65)	(2.39, 4.23)	
		(↗↘)	(9.9, 54.1)	(13.5, 39.4)		(7.10, 50.7)	(28.7, 38.09)	(34.8, 54.8)	(25.4, 67.8)	
	PB	(↑↓)	(.08, 26.8)	(.1, 41.8)		(0.04, 77.8)	(0.17, 35.4)	(0.52, 5.82)	(1.19, 0.69)	
		(↗↘)	(3.5, 69.5)	(5.9, 52.8)		(1.19, 20.9)	(8.33, 56.1)	(27.3, 66.3)	(50.3, 47.8)	
	SD	(↑↓)	(.8, 16.9)	(7.3, 19.8)		(0.18, 24.6)	(11.4, 27.1)	(1.71, 0.6)	(0.93, 5.64)	
		(↗↘)	(18.9.63.2)	(30.6, 42.2)		(15.4, 59.7	(28.4, 32.9)	(27.3, 40.3)	(27.6, 65.7)	
NDVI & SR (W/m^2^)	KPK	(↑↓)	(2.24, 49.5)	(0.64, 48.1)		(12.2, 47.4)	(4.67, 11.8)	(0.35, 21.6)	(12.1, 14.2)	
		(↗↘)	(22.01, 26.2)	(11.1, 40.1)		(17.1, 23.2)	(14.4, 69.1)	(18.1, 59.8)	(31.1, 42.6)	
	AJK	(↑↓)	(2.11, 45.9)	(0.29, 41.08)		(25.4, 33.9)	(0.35, 17.5)	(0.05, 58.4)	(8.77, 26.70)	
		(↗↘)	(32.9, 19.1)	(11.06, 47.5)		(16.6, 23.9)	(19.2, 62.8)	(13.7, 27.7)	(26.9, 37.6)	
	GB	(↑↓)	(1.88, 7.21)	(0.27, 11.3)		(23.6, 2.36)	(8.89, 3.42)	(0.35, 5.68)	(24.01, 0.34)	
		(↗↘)	(40.7, 50.1)	(23.6, 64.6)		(49.0, 24.9)	(43.5, 44.1)	(24.0, 69.9)	(62.2, 13.3)	
	BL	(↑↓)	(0.12, 85.4)	(0.29, 69.9)		(0.16, 50.3)	(1.15, 49.6)	(0.23, 6.20)	(9.15, 1.99)	
		(↗↘)	(1.43, 13.1)	(4.06, 25.6)		(4.76, 44.6)	(14.3, 34.8)	(17.6, 75.9)	(69.00, 19.8)	
	PB	(↑↓)	(0.19, 80.7)	(0.24, 52.9)		(0.09, 84.9)	(0.08, 34.9)	(0.03, 40.4)	(5.66, 5.56)	
		(↗↘)	(1.99, 17.0)	(4.52, 42.2)		(1.48, 13.5)	(5.82, 59.1)	(5.90, 53.6)	(43.4, 45.3)	
	SD	(↑↓)	(1.09, 70.9)	(3.01, 57.0)		(0.62, 30.1)	(2.41, 46.7)	(1.79, 40.5)	(5.85, 3.80)	
		(↗↘)	(7.31, 20.6)	(15.4, 24.5)		(8.7, 60.5)	(14.5, 36.2)	(13.4, 44.2)	(65.6, 24.7)	

(↑↓) Significant, (↗↘) non-significant (increase and decrease).

In PB (GS) reflects a negligible significant change in NDVI in response to temperature (only 0.1% of the area), highlighting the potential adaptation of local vegetation to temperature variations or the overriding impact of other factors such as irrigation practices (Fig. 7E). While in BL, a contrast is seen with an area of 0.22% showing significant annual NDVI responses to temperature, reinforcing the notion that in arid regions like BL, factors other than temperature might be driving vegetation health. In spring, GB shows minimal significant responses to temperature, with only 1.78% affected area, compared to AJK where the significant response is higher (19.7%) (Fig. 7H). This suggests spring warming benefits vegetation more in temperate, moisture-rich areas like AJK than in the colder, drier GB. PB shows a higher significant positive reaction to temperature changes in 77.8% of the area in summer, indicative of heat-adapted vegetation or a reflection of summer monsoons' critical role in the region (Fig. 7J–L). Annually, SD shows a small percentage (0.8%) of the area with significant positive NDVI responses to temperature. This minimal response underscores the potential saturation of temperature effects or the dominance of other limiting factors for vegetation, such as water availability. BL is unique, with a vast area showing non-significant reactions (21.6% annually) to SR, possibly indicating a saturation point where additional solar input does not translate to increased vegetative growth due to limiting factors like water scarcity. Precipitation significantly impacts the NDVI in all regions of Pakistan and considerably influences the annual vegetation dynamics. Temperature has varying effects, with the most notable impacts observed in GB, while it has a lesser influence in areas like PB. Conversely, SR has limited significant positive effects on NDVI, particularly in arid zones such as BL, implying that other factors may mediate vegetation response to solar inputs.

**Figure 8:**
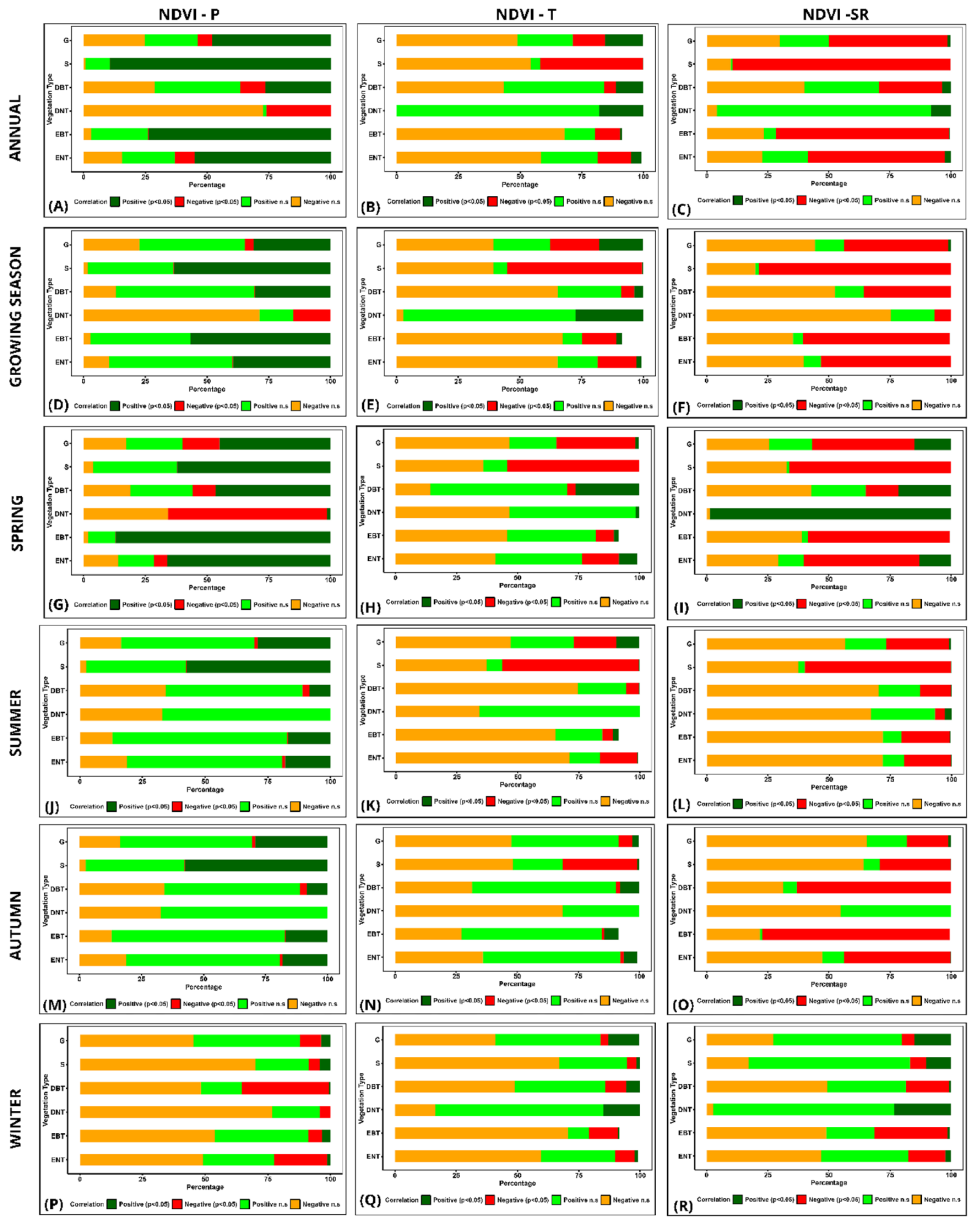
Spatial distribution of area percentage derived from partial correlation coefficients between NDVI and climatic variables across different vegetation types in Pakistan. Annual (**A**–**C**), growing season (**D**–**F**), Spring (**G**–**I**), summer (**J**–**L**), autumn (**M**–**O**), and winter (**P**–**R**).

The study illustrates a correlation between NDVI and climatic variables across different vegetation types, providing insights into ecological dynamics influenced by climate. Annually, ENT exhibits a significant growth increase of 54.9%, correlated with precipitation Fig. 6A. However, only 1.3% demonstrate a positive response during winter, while 21.2% are negatively affected by arid conditions (Figure S2). EBT shows an 87.1% positive response to spring precipitation, coinciding with their peak growth phase, which indicates their strong adaptation to seasonal precipitation patterns. In contrast, DBT presents a balanced spring response (Fig. 7G–I), with 46.5% showing significant growth (Fig. 8G–I); however, they suffer a 34.9% decrease in winter, highlighting their sensitivity to seasonal water changes.

Shrubs, adapted to arid environments, exhibit a considerable annual growth response of 89.3% to precipitation, emphasizing their dependence on precipitation for survival and growth (Fig. 8D–F). Nevertheless, this response drastically falls to 4.1% during winter (Fig. 8P–R), underlining the pronounced effect of seasonal water availability. Grasses, known for their resilience, show a 48% positive annual response to precipitation, indicating moderate dependence on water (Table S3). However, they experience a 14.9% positive growth response in winter and autumn (Fig. 8M–O), contrasting with the higher adverse reactions in other seasons, which suggests their unique adaptation to cooler, potentially wetter winters. Regarding SR, ENT experiences a 56.1% negative response annually, indicating potential damage from excessive light or heat (Fig. 8A–C). Yet, their winter adaptation reveals a reduced, yet significant, 15.1% negative response, suggesting lesser but ongoing stress from lower levels of winter sunlight. Conversely, grasses exhibit a balanced response to solar radiation, with significant positive responses more evident in winter at 14.9%, reflecting their inherent resilience and adaptability to various light conditions (Fig. 8J–L).

### Cross wavelet transform of NDVI and climatic variables across Pakistan: regional coherence and phase relationships (2000–2023)

**Figure 9:**
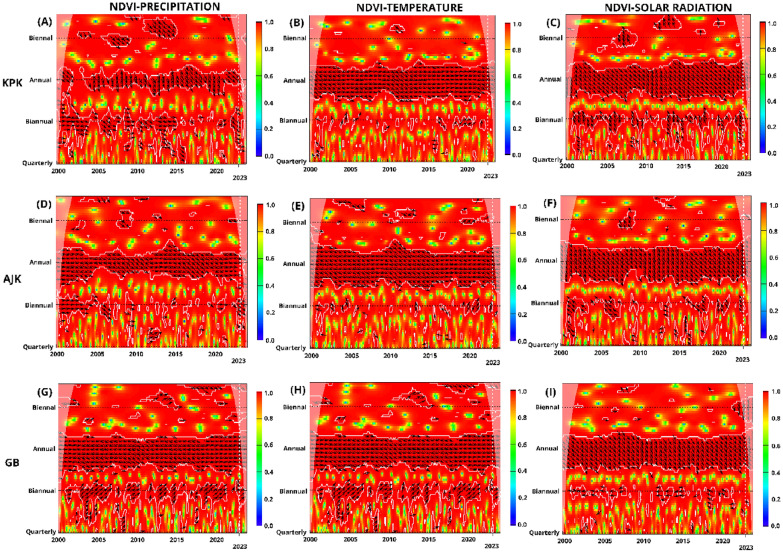
CWT analysis of NDVI with precipitation, temperature, and solar radiation in Pakistan segmented by regions: KPK, AJK, and GB. Panels (**A**–**C**) NDVI and precipitation, (**D**–**F**) NDVI and temperature, and (**G**–**I**) NDVI and solar radiation. *Note*: Color gradients from red to yellow signify increasing wavelet power, marking significant coherence areas. Contours indicate statistical significance (p < 0.05), with a cone of influence showing potential edge distortions. The y-axis shows periods from quarterly to biennial, and the x-axis covers 2000 to 2023.

The Cross Wavelet Transform (CWT) analysis conducted across various regions of Pakistan (KPK, AJK, GB, BL, PB, and SD) provides a complex view of the relationship between NDVI and climatic variables (Precipitation, Temperature, and SR) from 2000 to 2023. This analysis emphasizes significant segments of coherence and critical periods of phase relationships, offering an understanding of the complex relationship between vegetation health and environmental factors. KPK, reveals a continuous and significant segment of coherence between NDVI and precipitation on an annual scale, presenting as a prolonged coherent interval characterized by an extended periodicity. This segment is characterized by the direction of arrows, indicating a phase-in condition where NDVI follows precipitation with a lag, suggesting precipitation as a key driver of vegetation health (Fig. 9A–C). A similar coherence pattern is observed between NDVI and Temperature and NDVI and SR, with significant strong relationships at annual scales. These findings suggest that temperature and SR positively correlate with NDVI after an initial response period, albeit with a delayed effect (Fig. 9G–I). AJK exhibits similar coherence and phase patterns as KPK, reinforcing that these neighboring regions share comparable climatic influences on vegetation (Fig. 9D–F). For GB, the CWT analysis highlights a subtle interaction, particularly with precipitation, portraying a phase-out relationship at annual scales from 2004 to 2023. This pattern suggests a complex or potentially inverse relationship, where increases in precipitation do not directly correlate with improvements in vegetation health, likely due to the region's distinctive climatic and geographical attributes.

**Figure 10:**
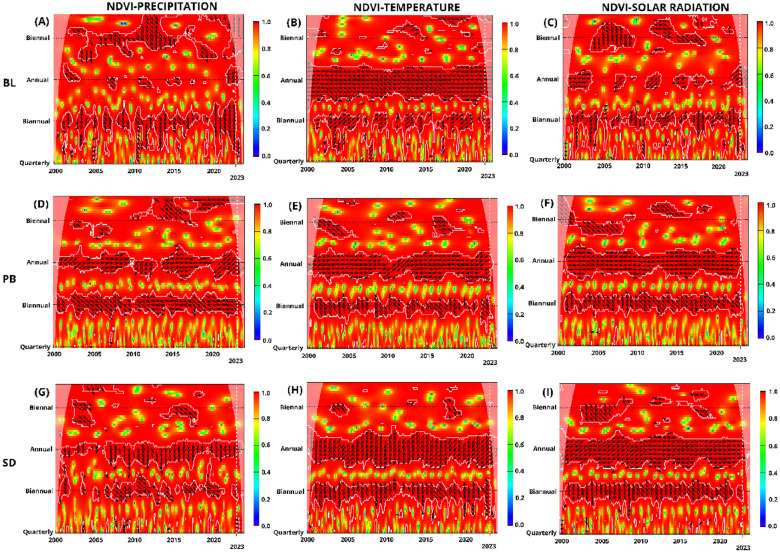
CWT Analysis of NDVI in relation to precipitation, temperature, and solar radiation in Pakistan segmented by regions: Balochistan (BL), Punjab (PB), and Sindh (SD). Panels (**A**–**C**) NDVI and precipitation, (**D**–**F**) NDVI and temperature, and (**G**–**I**) NDVI and solar radiation. *Note*: Color gradients from red to yellow signify increasing wavelet power, marking significant coherence areas. Contours indicate statistical significance (p < 0.05), with a cone of influence showing potential edge distortions. The y-axis shows periods from quarterly to biennial, and the x-axis covers 2000 to 2023.

In BL, temperature and SR show an in-phase, lagged influence on NDVI between quarterly and biannual scales, indicating delayed vegetation responses to climatic changes. However, at an annual scale, the observed inverse phase relationships, marked by arrows pointing downward to the left, signify that higher temperatures and increased solar radiation may adversely affect vegetation health, reflecting potential stress conditions such as heat stress or drought (Fig. 10D–F) (Fig. 10G–I). For precipitation, distinct periods (2001–2004, 2006–2009, 2012–2014) showcase an inverse relationship with NDVI, depicted by arrows pointing upwards to the left, hinting at a complex or delayed vegetation response to precipitation (Fig. 10A–C). However, in 2023, this trend shifts, with vectors pointing downward to the left in a significant pattern, suggesting an evolving adverse impact of precipitation on vegetation, possibly due to altered precipitation patterns or other environmental changes. For PB, the scenario diverges; annual analysis from 2000 to 2023 illustrates a consistent inverse relationship, potentially signaling detrimental effects of excessive precipitation on vegetation.

Between quarterly and biannual periods, a positive lagged relationship between precipitation and NDVI in PB after an interval following precipitation typically increases NDVI, reflecting a beneficial effect of precipitation on vegetation after an unavoidable delay. In contrast, across annual, quarterly, and biannual scales, NDVI-Temperature and SR demonstrate a delayed negative relationship, indicating that decreases typically follow increases in temperature and solar exposure in vegetation health after a certain period. In SD, similar to PB, an annual lagged positive correlation has been observed between NDVI and precipitation, suggesting that intermittent precipitation events can enhance vegetation growth. In contrast, temperature and SR depicted an antiphase or negative impact, signifying that higher temperatures and increased SR typically correlate with decreased vegetation health, highlighting the challenges posed by heat and light stress in warmer areas of Pakistan. Hence, CWT analyses across Pakistan's regions illustrate a complex mosaic of vegetative responses to climatic variables, marked by direct and lagged correlations, phase-ins, and phase-outs. The observed patterns underscore the critical influence of regional climatic dynamics on vegetation health, highlighting the need for tailored environmental management strategies that consider these complex, localized ecological responses.

## Discussion

From 2000 to 2023, this study utilized NDVI derived from Landsat and climatic drivers to systematically examine the spatiotemporal variability of vegetation cover and the related climate-driven mechanisms in Pakistan at various temporal scales. Different regions of Pakistan experienced significant annual increases in NDVI and precipitation, with minor declines in temperature and decreases in SR. These findings are consistent with the research conducted by^99^, who noted increased monsoon, pre-monsoon, and annual precipitation along the China-Pakistan economic corridor from 1980 to 2016. The increase in precipitation at a rate of 0.4801 mm/yr indicates a more extensive climate change impact on the area.

The increased NDVI indicates better vegetation health and productivity, identified through satellite-based rainfall assessments, and its relationship with biomass productivity in arid regions, which emphasizes the contribution of precipitation to vegetative growth^100^. Furthermore, the changes in precipitation that occur in the various areas and fluctuate seasonally affirm the environmental changes^101,102^. Our investigation revealed that Pakistan experienced an annual and seasonal increase in NDVI, with AJK, KPK, and PB showing significant greening trends in 57.5%, 45.7%, and 81.2% of their areas, respectively. These findings are consistent with the work of^103^, who stated a significant change in land use in Southern Punjab, and^26^ examined variability in precipitation among regions, indicating a correlation between these factors and the observed NDVI dynamics. The extensive vegetation growth in Punjab is similar to the patterns observed in the Lodhran District, supporting the influence of land use changes on regional greening trends^67,104^. The areas showing positive changes in GB and BL were more subtle, with 8.3% and 44.7% respectively. These changes correspond with environmental challenges such as land degradation and highlight the diverse ecological responses across different regions^105^. Seasonal analyses further revealed disparities, with AJK and KPK leading in vegetation increases during the growing season (GS) at 60.1% and 54.0%, respectively^106^. Additionally, the observed significant increases in NDVI in KPK and PB can be contextualized by the localized adaptation strategies to climate change^107,108^.

Our research indicates that different vegetation types respond differently to climatic variables, consistent with global trends in similar ecosystems. The ENT showed a significant annual growth increase of 54.9%, mainly due to the influence of precipitation. This aligns with^109^ findings, who observed similar vegetation responses to climate variations across different biomes and seasons. The contrast between the spring growth of EBT and the pronounced winter decline in DBT highlights the complex relationships between vegetation types and seasonal climate patterns. This is similar to the ecological dynamics explored by Jihua Zhou et al. (2016) in alpine and semi-arid regions. Additionally, the adaptability of shrubs and grasses to varying climatic conditions is noteworthy, with shrubs displaying an annual growth response of 89.3% to precipitation and grasses exhibiting a unique winter growth response. This underscores the diverse survival strategies among vegetation types, as Zhang et al. (2017) and Zhuang et al. (2020) noted. Finally, the negative response of ENT to SR annually, coupled with their reduced winter stress, suggests a complex interaction between light exposure and vegetation health^110^.

In KPK and AJK, precipitation significantly influences NDVI, suggesting water availability is a critical driver of vegetation dynamics. In contrast, temperature is dominant in Gilgit-Baltistan (GB) BL demonstrates a strong dependency on precipitation, aligning with expectations for arid climates, while Punjab (PB) and Sindh (SD) exhibit varying degrees of sensitivity to climatic inputs, reflecting the intricate balance between water availability, temperature, and SR^7,30^. Particularly, the minimal significant response to temperature in SD underscores the potential limitations imposed by other factors, such as water scarcity. The diverse impacts of SR across regions, with negligible positive responses in KPK and AJK versus significant adverse effects in BL, highlight the importance of considering local environmental conditions and adaptive mechanisms in understanding vegetation responses^16,105,111^.

The CWT analysis showed significant coherence between NDVI and various climatic variables across different regions in Pakistan, mirroring patterns observed in other studies. For instance, the phase-in condition between NDVI and precipitation in KPK, with NDVI following precipitation with a lag, supports findings by^112,113^, indicating precipitation as a crucial driver for vegetation health. This alignment suggests a widespread climatic influence on vegetation, reinforcing the results of similar patterns in AJK. A significant segment of coherence between NDVI and temperature and NDVI and SR, with delayed effects, aligns with the shifts^114–116^. These studies affirm that other climatic factors, beyond precipitation, significantly impact NDVI dynamics, aligning with our findings across Pakistan’s regions. Smaller-scale seasonal variations and local circulation across BL, PB and SD correspond to a global pattern where localized environmental conditions, such as irrigation practices or land use changes, affect vegetation health^117^. GB's minimal significant changes across all variables and seasons suggest a unique ecological resilience or indifference, possibly due to its high altitude and rugged terrain impacting local climate-vegetation relationships distinctly from the rest of Pakistan. The CWT found that the impact of temperature and SR on vegetation in all regions was dual. At shorter scales, an immediate increase in these factors may have a stressful effect on vegetation, as evidenced by the inverse relationship between them. However, when assessed annually, the positive correlation between the two factors, even with a lag, suggests beneficial effects, likely resulting from extended growing seasons or enhanced photosynthetic efficiency. These findings emphasize the importance of considering the temporal scales and environmental context when evaluating the impact of climatic variables on vegetation health. Pakistan's response to climatic variables regarding vegetation is regionally specific and varies with seasons. The changes in NDVI are mainly driven by precipitation and temperature annually and during the growing season. However, the non-significant responses observed across all regions during different seasons highlight the complexity of ecological interactions. The spatial and temporal heterogeneity emphasizes the relationship between climate factors and vegetation health, suggesting environmental management and agricultural practices across the diverse landscapes of Pakistan.

## Conclusion

In the current investigation, the influence of various regional environmental parameters, particularly NDVI, precipitation, temperature, and SR, was analyzed to assess the dynamic interactions influencing vegetation health across Pakistan. Our results clearly distinguish the vegetative response to climatic variabilities, characterized by an average national NDVI of 0.2345 ± 0.139, reflecting moderate vegetation vitality influenced by diverse regional climates. The quantitative analysis from linear regression models demonstrated an annual increase in NDVI of 0.00197 per year (p < 0.0001), accompanied by a statistically significant rise in precipitation by 0.4801 mm/year (p = 0.0016). The Mann–Kendall trend tests further supported these findings, reinforcing the positive trend in vegetation health across the study period. The region of Kashmir emerged as a rich vegetation area, having the highest NDVI value of 0.433 ± 0.023, due to its significant precipitation average of 1119.07 ± 157.45 mm. BL showed a different pattern from the rest. It had a low NDVI of 0.102 ± 0.01. This is probably because its dry average precipitation of 163.75 ± 46.86 mm underscores the significance of hydrothermal conditions in the local vegetation dynamics. The Cross Wavelet Transform analysis provided valuable insights into the coherence between NDVI and climatic variables over time and space. Significant coherence periods where NDVI closely follows precipitation patterns were identified, with notable phase lags indicating the temporal response of vegetation to climatic inputs. The analysis conducted using the CWT technique from 2000 to 2023 provided valuable insights into the coherence between NDVI and climatic variables over time and space. The correlation between NDVI and yearly precipitation was prominent in regions like KPK and AJK, emphasizing the crucial role rainfall plays in preserving the well-being of vegetation. A considerable 45.7% of KPK's land area displayed a substantial upsurge in plant growth, indicating a significant enhancement in ecological conditions. Our study explored into the spatial and temporal dynamics of vegetation health, observing distinct regional variations where provinces like Punjab and Khyber Pakhtunkhwa exhibited robust vegetation responses compared to more arid regions like Balochistan. This underscores the variable impact of climatic factors across different geographical areas. Our findings suggest that BL's arid and semi-arid regions had a relatively lower response, with only 8.3% of the land showing positive annual changes. This highlights the complex relationship between vegetation and extreme climatic conditions. Climate change produces diverse effects in different regions and seasons. For example, Punjab has displayed remarkable adaptability to seasonal changes, with over 81% of its land showing significant increases in vegetation. The health of regional vegetation is heavily influenced by monsoon patterns, with regions like KPK and AJK experiencing a substantial increase in vegetation growth during the monsoon season. The ecological insights provided by our analysis, such as the enhanced vegetation growth in northern regions during monsoon seasons and the interaction between vegetation health and climatic factors like solar radiation and temperature, are vital for formulating region-specific conservation strategies. By implementing sophisticated methods such as the Cross Wavelet Transform, we have comprehensively understood the intricate interrelationships between climatic patterns and vegetation growth over extended periods. These insights have significant implications for environmental management and agricultural practices, particularly in regions that share similar climatic conditions. To enhance sustainability and ecological resilience, we recommend the adoption of adaptive management strategies that are informed by continuous monitoring of climatic trends and vegetation responses. Policymakers should consider integrating advanced technologies such as machine learning to forecast changes and devise responsive strategies effectively. Furthermore, expanding research to encompass a broader range of climate variables will further refine our understanding and support the development of targeted interventions. These proactive measures are crucial for optimizing land use and ensuring the long-term viability of agricultural systems in affected areas.

### Supplementary Information

Supplementary Information.

## Data Availability

Data is provided within the manuscript or supplementary information files.
