# Supplementary material for: Analyzing vegetation health dynamics across seasons and regions through NDVI and climatic variables
Source: Sci Rep. 2024 May 23;14:11775. doi: 10.1038/s41598-024-62464-7 (PMC11116382; doi:10.1038/s41598-024-62464-7)
Supplement: Supplementary file 1 — Supplementary Information. [file 41598_2024_62464_MOESM1_ESM.docx]

**Supplementary Materials**


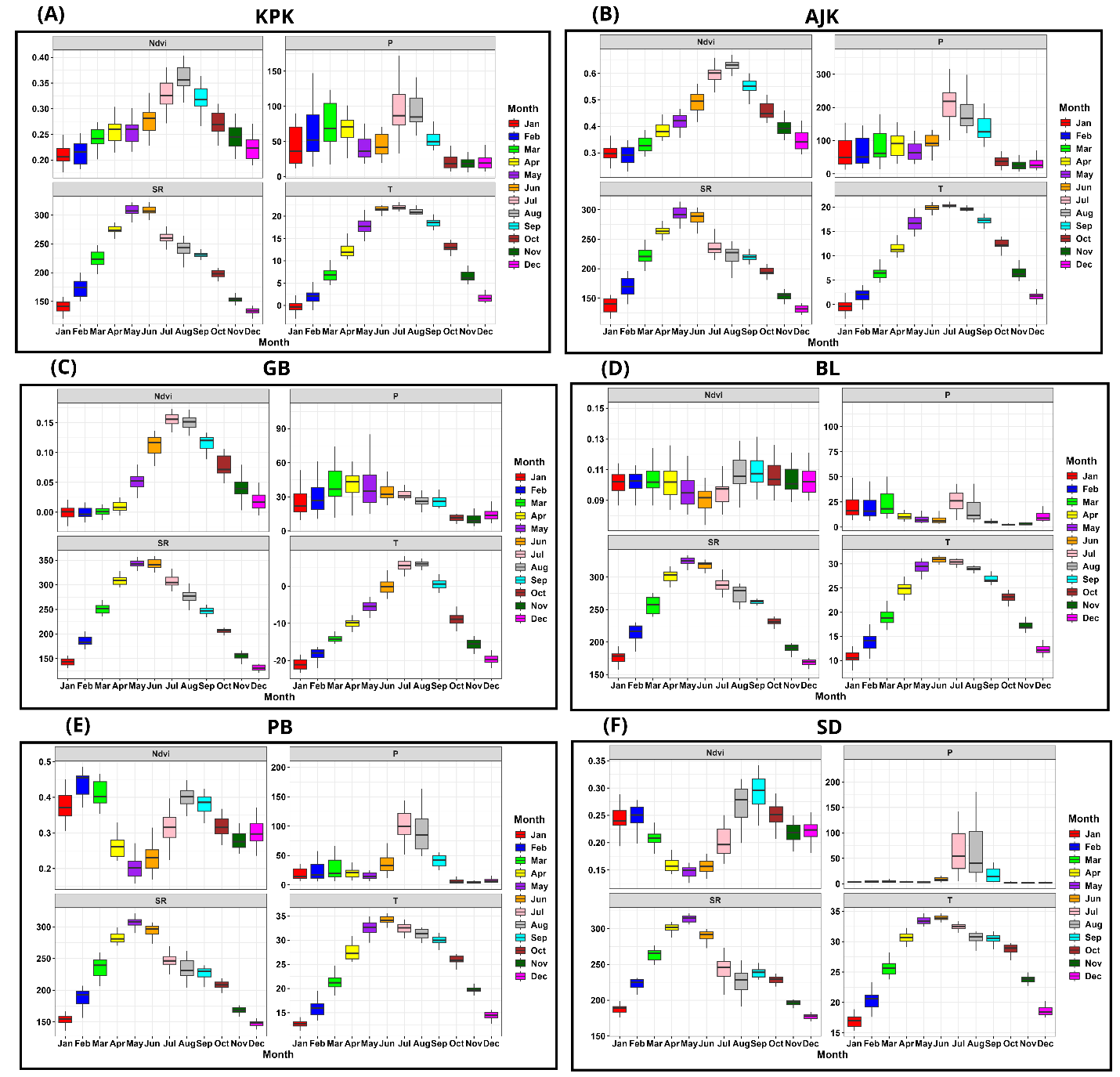


**Figure S1: Temporal Trends of Mean Normalized Difference Vegetation Index (NDVI) and Associated Climatic Variables Across Pakistan, 2000-2023.** This figure presents the interannual fluctuations and comparative analysis of the mean NDVI and key climatic drivers, including Temperature, Precipitation, and Solar Radiation (SR), across different regions of Pakistan. Each panel represents a specific province, illustrating distinct environmental conditions and vegetation responses over the twenty-three-year period: (A) Khyber Pakhtunkhwa (KPK), (B) Azad Jammu and Kashmir (AJK), (C) Gilgit-Baltistan (GB), (D) Baluchistan (BL), (E) Punjab (PB), and (F) Sindh (SD). The graphical representation aims to provide insights into the complex dynamics between vegetation health and climatic influences within each geographical locale.


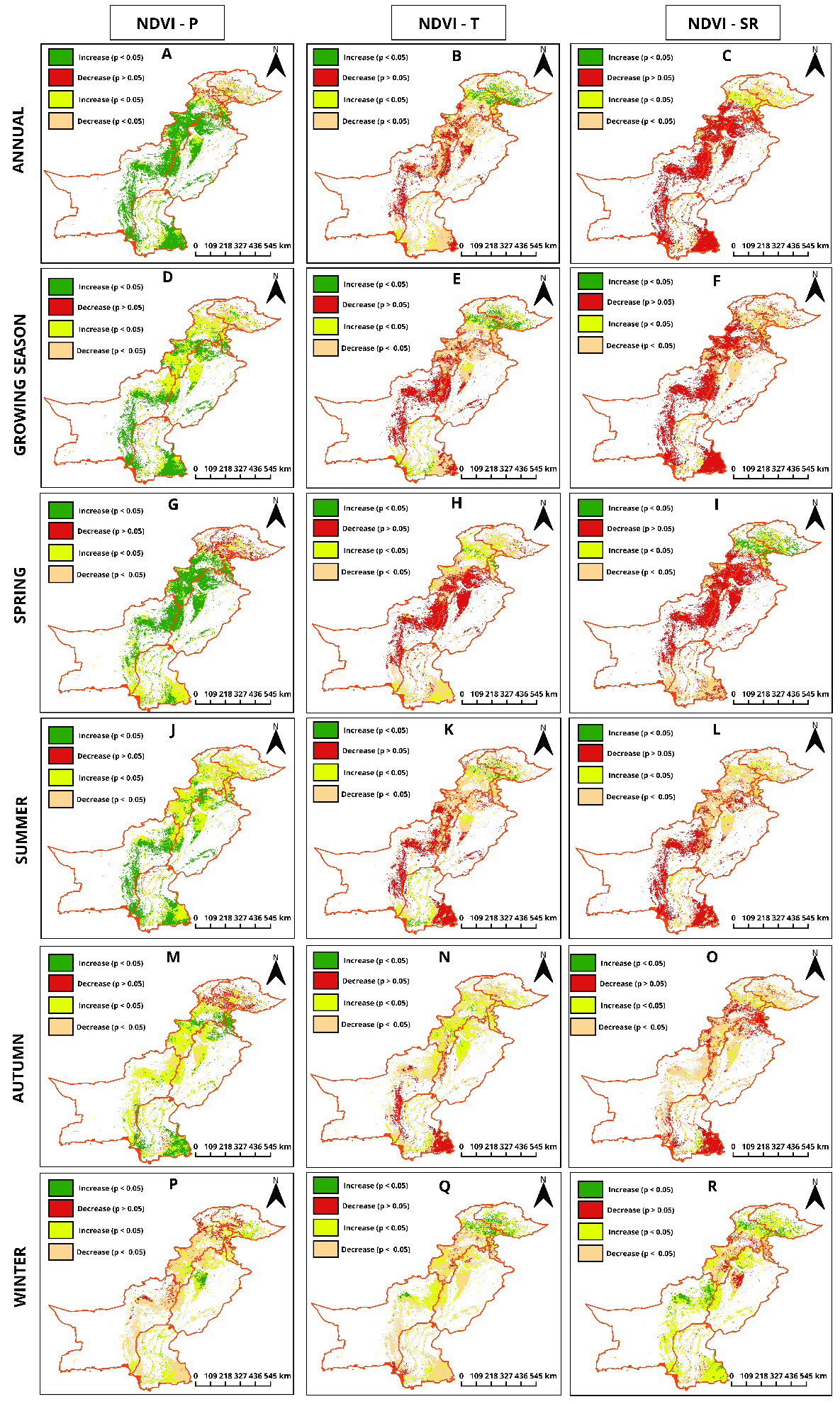


**Figure S2: Spatial Distribution of Partial Correlation Coefficients between NDVI and Climatic Variables across different Vegetation Types in Pakistan. The correlation between NDVI and precipitation, temperature, and solar radiation (SR) is represented across different temporal scales: Annual (A–C), Growing Season (D–F), Spring (G–I), Summer (J–L), Autumn (M–O), and Winter (P–R). Each color represents different threshold of significance level (%).**

**Table S3: Percentage of Area Exhibiting NDVI Responses to Climatic Variables Across Various Vegetation Types in Pakistan**

| Parameters | Vegetation Types | Correlation | Annual | GS | Spring | Summer | Autumn | Winter |
| --- | --- | --- | --- | --- | --- | --- | --- | --- |
| NDVI & P | ENT | (↑) | 54.9 | 39.4 | 66.1 | 18.1 | 44.2 | 1.3 |
|  |  | (↓) | 8.3 | 0.3 | 5.3 | 1.1 | 3.5 | 21.2 |
|  |  | (↗) | 21.1 | 50 | 14.6 | 61.9 | 42.9 | 28.3 |
|  |  | (↘) | 15.7 | 10.2 | 13.9 | 18.8 | 9.3 | 49.2 |
|  | EBT | (↑) | 73.9 | 56.6 | 87.1 | 16.9 | 80.6 | 3.4 |
|  |  | (↓) | 0.1 | 0 | 0 | 0.4 | 0 | 5.5 |
|  |  | (↗) | 23.1 | 40.6 | 11 | 69.8 | 18.5 | 37.3 |
|  |  | (↘) | 2.9 | 2.8 | 1.9 | 12.9 | 0.9 | 53.9 |
|  | DBT | (↑) | 26.4 | 30.6 | 46.5 | 8.4 | 48.3 | 0.5 |
|  |  | (↓) | 10.2 | 0.4 | 9.4 | 2.8 | 2 | 34.9 |
|  |  | (↗) | 34.8 | 55.9 | 25.1 | 54.4 | 37.6 | 16.3 |
|  |  | (↘) | 28.7 | 13.2 | 19 | 34.3 | 12 | 48.2 |
|  | S | (↑) | 89.3 | 63.5 | 62.1 | 57.5 | 24.1 | 4.1 |
|  |  | (↓) | 0.1 | 0.1 | 0.1 | 0.2 | 0.1 | 4.5 |
|  |  | (↗) | 9.7 | 34.8 | 34 | 39.7 | 62.7 | 21.3 |
|  |  | (↘) | 0.8 | 1.6 | 3.8 | 2.6 | 13.1 | 70 |
|  | G | (↑) | 48 | 31.2 | 45 | 29.1 | 14.5 | 3.8 |
|  |  | (↓) | 5.8 | 3.5 | 14.9 | 1.2 | 13.9 | 8.5 |
|  |  | (↗) | 21.3 | 42.8 | 22.8 | 53.2 | 46.9 | 42.4 |
|  |  | (↘) | 24.9 | 22.5 | 17.3 | 16.5 | 24.8 | 45.3 |
| NDVI & T | ENT | (↑) | 4.1 | 1.9 | 7.6 | 0.1 | 5.5 | 1.5 |
|  |  | (↓) | 13.5 | 15.8 | 15.1 | 15.4 | 1.4 | 7.7 |
|  |  | (↗) | 23.1 | 16.2 | 35.5 | 12.5 | 56.3 | 30.3 |
|  |  | (↘) | 58.5 | 65.2 | 41.1 | 71.1 | 35.8 | 59.6 |
|  | EBT | (↑) | 1 | 2.2 | 1.8 | 2.4 | 5.7 | 0.5 |
|  |  | (↓) | 9.9 | 14.1 | 7.5 | 4.3 | 1 | 11.7 |
|  |  | (↗) | 12.5 | 7.9 | 36.2 | 19.2 | 57.5 | 8.6 |
|  |  | (↘) | 68 | 67.2 | 45.8 | 65.4 | 27.2 | 70.5 |
|  | DBT | (↑) | 11.1 | 3.7 | 26 | 0.3 | 7.9 | 5.6 |
|  |  | (↓) | 4.8 | 5.1 | 3.5 | 5.1 | 1.7 | 8.6 |
|  |  | (↗) | 40.5 | 25.9 | 56.1 | 19.9 | 58.8 | 36.9 |
|  |  | (↘) | 43.5 | 65.2 | 14.3 | 74.6 | 31.6 | 48.8 |
|  | S | (↑) | 0.1 | 0.5 | 0 | 0.5 | 0.8 | 1.3 |
|  |  | (↓) | 41.6 | 54.5 | 54 | 55.7 | 30.5 | 4.1 |
|  |  | (↗) | 3.9 | 5.6 | 9.8 | 6.6 | 20.5 | 27.5 |
|  |  | (↘) | 54.4 | 39.3 | 36 | 37.1 | 48.1 | 67 |
|  | G | (↑) | 15.3 | 17.6 | 1.5 | 9.2 | 2.6 | 12.7 |
|  |  | (↓) | 13.2 | 19.9 | 32.2 | 17.5 | 5.6 | 3.3 |
|  |  | (↗) | 22.4 | 22.9 | 19.4 | 25.8 | 43.8 | 42.7 |
|  |  | (↘) | 48.9 | 39.3 | 46.6 | 47.2 | 47.7 | 41 |
| NDVI & SR | ENT | (↑) | 2.5 | 0.1 | 13.1 | 0.2 | 0.3 | 2.4 |
|  |  | (↓) | 56.1 | 53.2 | 47.1 | 19.3 | 43.5 | 15.1 |
|  |  | (↗) | 18.7 | 6.9 | 10.5 | 8.6 | 9 | 35.7 |
|  |  | (↘) | 22.7 | 39.8 | 29.2 | 72 | 47.3 | 46.8 |
|  | EBT | (↑) | 0.3 | 0.1 | 0.2 | 0.3 | 0 | 0.9 |
|  |  | (↓) | 70.9 | 60.1 | 58 | 19.9 | 76.7 | 30.1 |
|  |  | (↗) | 4.9 | 4 | 2.5 | 7.4 | 1 | 19.6 |
|  |  | (↘) | 23.3 | 35.3 | 38.9 | 72 | 21.7 | 48.9 |
|  | DBT | (↑) | 3.5 | 0.1 | 21.6 | 0.2 | 0 | 0.9 |
|  |  | (↓) | 25.8 | 35.5 | 13.4 | 12.8 | 63.2 | 17.6 |
|  |  | (↗) | 30.8 | 11.8 | 22.3 | 16.8 | 5.6 | 32.1 |
|  |  | (↘) | 39.9 | 52.6 | 42.7 | 70.2 | 31.2 | 49.4 |
|  | S | (↑) | 0 | 0.1 | 0 | 0.1 | 0.1 | 10.3 |
|  |  | (↓) | 89.5 | 78.5 | 66 | 59.7 | 29 | 6.5 |
|  |  | (↗) | 0.6 | 1.5 | 1.2 | 2.7 | 6.7 | 66.1 |
|  |  | (↘) | 9.8 | 19.8 | 32.6 | 37.4 | 64.2 | 17 |
|  | G | (↑) | 1.2 | 0.9 | 15 | 1 | 1 | 14.9 |
|  |  | (↓) | 48.8 | 42.8 | 41.9 | 25.7 | 16.9 | 5.2 |
|  |  | (↗) | 20.2 | 11.8 | 17.4 | 16.6 | 16.5 | 52.6 |
|  |  | (↘) | 29.7 | 44.4 | 25.6 | 56.6 | 65.4 | 27.2 |

(↑) Significant Increase (↓) Significant Decrease (↗) Insignificant Increase (↘) Insignificant Decrease

**Table S4.** Presents the distribution of statistical significance levels (P < 0.01, P < 0.05, P < 0.10, and P > 0.10) for various regions and seasons, including annual (**S4**A) and season data as GS (**S4**B), Spring (**S4**C), Summer (**S4**D), Autumn (**S4**E) and Winter (**S4**F). It highlights positive (+) and negative (-) significance percentages across regions like KPK, AJK, GB, BL, PB, and SD.

**Table S4A: Annual Statistical Significance Levels (%)**

| **Significant Levels** | **Annual** | | | | | |
| --- | --- | --- | --- | --- | --- | --- |
|  | **KPK** | **AJK** | **GB** | **BL** | **PB** | **SD** |
| P < 0.01 (+) | 45.7 | 57.5 | 8.3 | 44.7 | 81.2 | 40.0 |
| P < 0.01 (-) | 0.6 | 0.1 | 0.2 | 0.8 | 1.1 | 5.3 |
| P < 0.05 (+) | 12.6 | 5.2 | 8.4 | 19.4 | 7.3 | 15.6 |
| P < 0.05 (-) | 0.3 | 0.2 | 0.3 | 0.6 | 0.4 | 2.4 |
| P < 0.10 (+) | 6.8 | 2.8 | 7.1 | 8.8 | 2.8 | 8.0 |
| P < 0.10 (-) | 0.3 | 0.3 | 0.5 | 0.5 | 0.3 | 1.6 |
| P > 0.10 (+) | 26.7 | 20.9 | 55.7 | 18.3 | 5.1 | 16.3 |
| P > 0.10 (-) | 7.1 | 13.0 | 19.4 | 7.0 | 1.8 | 10.8 |

**Table S4B: GS Statistical Significance Levels (%)**

| **Significant Levels** | **GS** | | | | | |
| --- | --- | --- | --- | --- | --- | --- |
|  | **KPK** | **AJK** | **GB** | **BL** | **PB** | **SD** |
| P < 0.01 (+) | 54.0 | 60.1 | 13.5 | 29.5 | 68.2 | 23.8 |
| P < 0.01 (-) | 0.7 | 0.3 | 0.4 | 0.9 | 1.1 | 2.7 |
| P < 0.05 (+) | 11.6 | 6.2 | 11.1 | 20.3 | 11.1 | 16.0 |
| P < 0.05 (-) | 0.4 | 0.3 | 0.5 | 0.6 | 0.6 | 1.9 |
| P < 0.10 (+) | 5.1 | 2.9 | 7.6 | 10.8 | 4.5 | 9.2 |
| P < 0.10 (-) | 0.3 | 0.4 | 0.5 | 0.5 | 0.4 | 1.3 |
| P > 0.10 (+) | 21.5 | 17.7 | 52.0 | 30.5 | 10.9 | 30.9 |
| P > 0.10 (-) | 6.4 | 12.2 | 14.4 | 7.0 | 3.2 | 14.2 |

**Table S4C: Spring Statistical Significance Levels (%)**

| **Significant Levels** | **Spring** | | | | | |
| --- | --- | --- | --- | --- | --- | --- |
|  | **KPK** | **AJK** | **GB** | **BL** | **PB** | **SD** |
| P < 0.01 (+) | 41.37 | 56.04 | 6.55 | 19.2 | 43.9 | 21.38 |
| P < 0.01 (-) | 0.7 | 0.26 | 0.25 | 0.4 | 0.9 | 2.46 |
| P < 0.05 (+) | 13.05 | 4.91 | 7.51 | 19.5 | 17.6 | 14.87 |
| P < 0.05 (-) | 0.6 | 1.14 | 0.60 | 0.4 | 0.6 | 1.67 |
| P < 0.10 (+) | 6.43 | 1.79 | 5.89 | 12.3 | 8.0 | 9.58 |
| P < 0.10 (-) | 0.66 | 1.53 | 0.79 | 0.4 | 0.4 | 1.22 |
| P > 0.10 (+) | 26.07 | 15.61 | 53.45 | 39.9 | 22.9 | 37.20 |
| P > 0.10 (-) | 11.08 | 18.71 | 24.96 | 7.8 | 5.6 | 11.62 |

**Table S4D: Summer Statistical Significance Levels (%)**

| **Significant Levels** | **Summer** | | | | | |
| --- | --- | --- | --- | --- | --- | --- |
|  | **KPK** | **AJK** | **GB** | **BL** | **PB** | **SD** |
| P < 0.01 (+) | 43.3 | 34.9 | 11.2 | 25.1 | 63.0 | 19.3 |
| P < 0.01 (-) | 0.6 | 0.4 | 0.4 | 0.8 | 0.7 | 2.7 |
| P < 0.05 (+) | 14.0 | 16.2 | 10.0 | 19.3 | 12.0 | 11.4 |
| P < 0.05 (-) | 0.5 | 0.5 | 0.6 | 0.5 | 0.6 | 1.8 |
| P < 0.10 (+) | 6.9 | 7.4 | 6.8 | 11.2 | 5.0 | 7.8 |
| P < 0.10 (-) | 0.4 | 0.6 | 0.7 | 0.4 | 0.5 | 1.3 |
| P > 0.10 (+) | 26.3 | 26.8 | 48.8 | 35.5 | 13.6 | 41.3 |
| P > 0.10 (-) | 8.1 | 13.2 | 21.5 | 7.2 | 4.7 | 14.3 |

**Table S4E: Autumn Statistical Significance Levels (%)**

| **Significant Levels** | **Autumn** | | | | | |
| --- | --- | --- | --- | --- | --- | --- |
|  | **KPK** | **AJK** | **GB** | **BL** | **PB** | **SD** |
| P < 0.01 (+) | 36.2 | 44.2 | 4.8 | 19.5 | 39.5 | 16.6 |
| P < 0.01 (-) | 0.7 | 0.2 | 0.2 | 0.9 | 3.6 | 4.7 |
| P < 0.05 (+) | 13.14 | 10.9 | 6.3 | 20.0 | 13.2 | 16.1 |
| P < 0.05 (-) | 0.56 | 0.2 | 0.4 | 0.8 | 2.4 | 2.8 |
| P < 0.10 (+) | 6.43 | 4.2 | 5.3 | 12.3 | 6.7 | 9.4 |
| P < 0.10 (-) | 0.48 | 0.3 | 0.6 | 0.6 | 1.5 | 1.8 |
| P > 0.10 (+) | 29.34 | 25.2 | 54.1 | 37.7 | 22.1 | 34.7 |
| P > 0.10 (-) | 13.75 | 14.7 | 28.3 | 8.2 | 11.0 | 14.0 |

**Table S4F: Winter Statistical Significance Levels (%)**

| **Significant Levels** | **Winter** | | | | | |
| --- | --- | --- | --- | --- | --- | --- |
|  | **KPK** | **AJK** | **GB** | **BL** | **PB** | **SD** |
| P < 0.01 (+) | 26.7 | 33.5 | 1.0 | 13.0 | 46.8 | 23.0 |
| P < 0.01 (-) | 0.9 | 0.2 | 0.7 | 0.6 | 1.4 | 5.2 |
| P < 0.05 (+) | 14.0 | 15.1 | 2.8 | 14.2 | 14.3 | 10.9 |
| P < 0.05 (-) | 1.1 | 0.7 | 2.4 | 0.6 | 0.9 | 2.1 |
| P < 0.10 (+) | 7.0 | 6.3 | 2.8 | 9.4 | 5.9 | 6.5 |
| P < 0.10 (-) | 0.9 | 0.5 | 2.2 | 0.5 | 0.6 | 1.1 |
| P > 0.10 (+) | 35.4 | 32.0 | 48.9 | 49.9 | 23.2 | 42.7 |
| P > 0.10 (-) | 14.0 | 11.8 | 39.2 | 11.8 | 7.0 | 8.6 |
